# Super-resolution microscopy enabled by high-efficiency surface-migration emission depletion

**DOI:** 10.1038/s41467-022-33726-7

**Published:** 2022-11-04

**Authors:** Rui Pu, Qiuqiang Zhan, Xingyun Peng, Siying Liu, Xin Guo, Liangliang Liang, Xian Qin, Ziqing Winston Zhao, Xiaogang Liu

**Affiliations:** 1 Centre for Optical and Electromagnetic Research, South China Academy of Advanced Optoelectronics, South China Normal University, Guangzhou, 510006 P. R. China; 2 National Center for International Research on Green Optoelectronics, Guangdong Engineering Research Centre of Optoelectronic Intelligent Information Perception, South China Normal University, Guangzhou, 510006 P. R. China; 3 MOE Key laboratory & Guangdong Provincial Key laboratory of Laser Life Science, South China Normal University, Guangzhou, 510631 P. R. China; 4 Department of Chemistry, National University of Singapore, 3 Science Drive 3, Singapore, 117543 Singapore; 5 Centre for BioImaging Sciences, National University of Singapore, 14 Science Drive 4, Singapore, 117557 Singapore; 6 Mechanobiology Institute, National University of Singapore, 5A Engineering Drive 1, Singapore, 117411 Singapore; 7 The N.1 Institute for Health, National University of Singapore, Singapore, 117456 Singapore

**Keywords:** Imaging techniques, Imaging and sensing, Super-resolution microscopy, Nanoparticles

## Abstract

Nonlinear depletion of fluorescence states by stimulated emission constitutes the basis of stimulated emission depletion (STED) microscopy. Despite significant efforts over the past decade, achieving super-resolution at low saturation intensities by STED remains a major technical challenge. By harnessing the surface quenching effect in NaGdF_4_:Yb/Tm nanocrystals, we report here high-efficiency emission depletion through surface migration. Using a dual-beam, continuous-wave laser manipulation scheme (975-nm excitation and 730-nm de-excitation), we achieved an emission depletion efficiency of over 95% and a low saturation intensity of 18.3 kW cm^−2^. Emission depletion by surface migration through gadolinium sublattices enables super-resolution imaging with sub-20 nm lateral resolution. Our approach circumvents the fundamental limitation of high-intensity STED microscopy, providing autofluorescence-free, re-excitation-background-free imaging with a saturation intensity over three orders of magnitude lower than conventional fluorophores. We also demonstrated super-resolution imaging of actin filaments in Hela cells labeled with 8-nm nanoparticles. Combined with the highly photostable lanthanide luminescence, surface-migration emission depletion (SMED) could provide a powerful mechanism for low-power, super-resolution imaging or biological tracking as well as super-resolved optical sensing/writing and lithography.

## Abstract (web summary)

The authors report high-efficiency emission depletion through a surface migration emission depletion mechanism, which takes advantage of the effects of surface quenching and energy migration in nanocrystals. They demonstrate super-resolution microscopy with very low depletion saturation intensities.

## Introduction

An energetically active solid surface is often involved in a variety of physical and chemical phenomena^1–3^. In particular, in the realm of nanoscale studies, nanoparticles with an enormous surface-to-volume ratio usually exhibit active, unique physical properties associated with the surface, such as surface quenchers^4,5^, surface states^6^, surface phonons^7^, surface plasmons^8^, and zigzag edge states^9^. Since surface energy quenchers or states often dominate the photoluminescence process^4^, enormous efforts have been made to suppress these side effects to improve emission performance^10,11^. Nevertheless, these surface phenomena can promote the exploitation of new mechanisms of optically controlled energy dissipation. For example, stimulated emission depletion (STED) microscopy offers a powerful tool for diffraction-breaking super-resolution imaging by attenuating luminescence emission^12–14^.

In conventional STED, spontaneous emission from fluorophores is turned off by stimulated emission to bring them to the off state^12,13^. In principle, the depletion saturation intensity of STED, defined as the input intensity at which the depletion efficiency reaches 50%, is inversely proportional to the lifetime of the emitting state and the stimulated emission cross-section^15–17^. Therefore, commonly used fluorescent probes with fast spontaneous emission may require high depletion intensity^14,18^, resulting in undesirable phototoxicity, photobleaching, or re-excitation background^19–22^. New strategies involving long-lived emission states of lanthanide ions have recently lowered the threshold for STED beam intensity^23–25^, but further improving the depletion performance of stimulated emission in this way means that the nanoprobes used must have both a larger cross-section and a longer lifetime. However, such ideal probe is not found in either inorganic luminescent materials or organic fluorophores. Moreover, long emission lifetime inevitably leads to slow imaging speed and low brightness. Therefore, it has always been of great importance to exploit new photophysical mechanisms to achieve efficient emission depletion while greatly reducing the saturation intensity^26,27^. The energy dissipation capability of surface quenchers may be stronger than stimulated emission, but actively manipulating surface quenching to switch off fluorescence remains a formidable challenge.

**Fig. 1:**
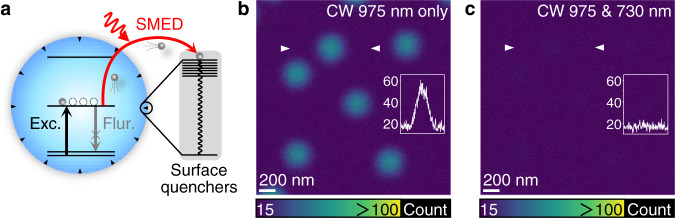
The surface-migration emission depletion (SMED) strategy enables optical depletion of NaGdF_4_:Yb/Tm nanoparticles. **a** The proposed SMED mechanism underlying emission depletion relies on an external energy source and bypasses fundamental constraints imposed by conventional STED microscopy. **b**, **c** Emission depletion of single NaGdF_4_:Yb/Tm (18/0.3%) nanoparticles under dual-beam CW laser manipulation (975-nm excitation and 730-nm de-excitation). Co-irradiation of the nanoparticles (~11 nm in diameter) on a glass slide with 975- and 730-nm continuous-wave (CW) lasers (*I*_975_ = 98 kW cm^−2^; *I*_730_ = 3.01 MW cm^−2^) significantly suppressed the 475-nm emission. Image dimensions: 800 × 800 pixels; pixel size: 3 nm; pixel dwell time: 200 μs. Images shown are averages of three repeated.

Here, we describe the experimental design of gadolinium-doped luminescent nanoparticles that can significantly reduce depletion intensity through surface-migration emission depletion (SMED) (Fig. 1a). The gadolinium sublattice provides a massive energy migration network to spatially and energetically direct energy transfer from the luminescent center to surface quenchers^4,10^. This effect facilitates the energy depletion of luminescent centers through nonradiative dissipation at nanoparticle surfaces, thereby enabling super-resolution imaging at low depletion saturation intensity.

## Results

### Surface migration enables high-efficiency emission depletion

**Fig. 2:**
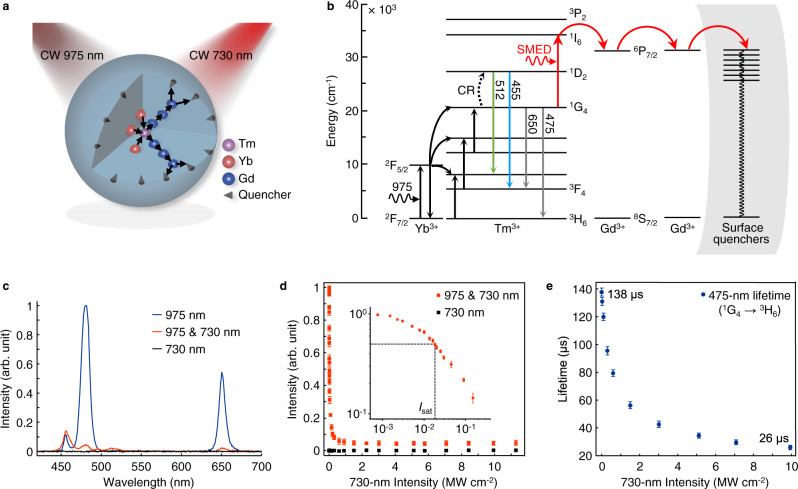
SMED-mediated high-efficiency emission depletion. **a** Schematic diagram of the SMED depletion process and energy transfer pathways in NaGdF_4_:Yb/Tm (18/0.3%) nanoparticles. **b** Proposed SMED mechanism of upconversion emissions from the ^1^G_4_ state of Tm^3+^. **c** Upconversion emission spectra of NaGdF_4_:Yb/Tm (18/0.3%) nanoparticles illuminated with different lasers (*I*_975_ = 21.7 kW cm^−2^; *I*_730_ = 3.01 MW cm^−2^). **d** Depletion intensity-dependence at a 975-nm excitation intensity of 21.7 kW cm^−2^. The inset in the log-log plot shows an enlarged view of the data in the low laser-intensity region, with a depletion saturation intensity of approximately 18.3 ± 0.9 kW cm^−2^. **e** Lifetime variation of the 475-nm emission versus the applied 730-nm CW depletion intensity (*I*_975_ = 72.1 kW cm^−2^). Data in **c**, **d** were presented as mean value ± standard deviation (SD). Error bars are defined as the SD of *n* = 3 independent measurements.

For proof of concept, we chose NaGdF_4_:Yb/Tm (18/0.3%) nanoparticles for single-particle imaging using a dual-laser optical microscopy system (Supplementary Fig. 1). Upon excitation with a 975-nm continuous-wave (CW) beam, these nanoparticles showed two main emission peaks at 475 and 650 nm, corresponding to the ^1^G_4_ → ^3^H_6_ and ^1^G_4_ → ^3^F_4_ transitions of Tm^3+^, respectively. When co-irradiated with a 730-nm CW beam, the Tm^3+^-activated nanoparticles showed almost complete emission depletion (Fig. 1b, c). This depletion process commences through excited-state absorption (ESA) to the ^1^G_4_ state of Tm^3+^, followed by energy pumping to the higher ^1^I_6_ state (Fig. 2a, b). Due to the overlap of energy levels between the ^1^I_6_ state of Tm^3+^ and the ^6^P_7/2_ state of Gd^3+^, efficient energy transfer subsequently occurs through energy migration from Tm^3+^ to surface defects or quenchers surrounding the Gd^3+^ ions^28,29^.

Spectroscopic characterizations indicated that the 475-nm emission from NaGdF_4_:Yb/Tm nanoparticles was depleted with over 95% efficiency upon co-irradiation with the 730-nm beam (Fig. 2c). On the contrary, emissions at 455 nm and 512 nm, assigned to the ^1^D_2_ → ^3^F_4_ and ^1^D_2_ → ^3^H_5_ transitions of Tm^3+^, respectively^30^, were slightly enhanced. This suggests the possibility of an energy transfer from the ^1^G_4_ state to the ^1^I_6_ state of Tm^3+^ and a subsequent ^1^I_6_ → ^1^D_2_ transition in the population of the ^1^D_2_ state. Intriguingly, depletion efficiency remains high within the depletion wavelength range of 715–745 nm, which facilitates laser selection and installation (Supplementary Fig. 2). At low depletion intensity, the depletion efficiency follows a sharp exponential decay (Fig. 2d). The measured depletion saturation intensity was 18.3 kW cm^−2^, almost 46 times lower than the 849 kW cm^−2^ reported in a previous attempt involving photon upconversion^25^ and more than three orders of magnitude lower than conventional fluorophores^17,19,31,32^. Since no emission is detectable with monochromatic excitation at 730 nm (Fig. 2c, d and Supplementary Fig. 2), the problem of re-excitation emission background inherent to conventional STED microscopy can be eliminated^22^. The occurrence of ESA was further supported by the shortened lifetime of the ^1^G_4_ state of Tm^3+^ upon depletion (Fig. 2e). The short emission lifetime at high depletion intensity can be ascribed to an increased transition rate of the ESA process.

### Mechanistic investigations of SMED

**Fig. 3:**
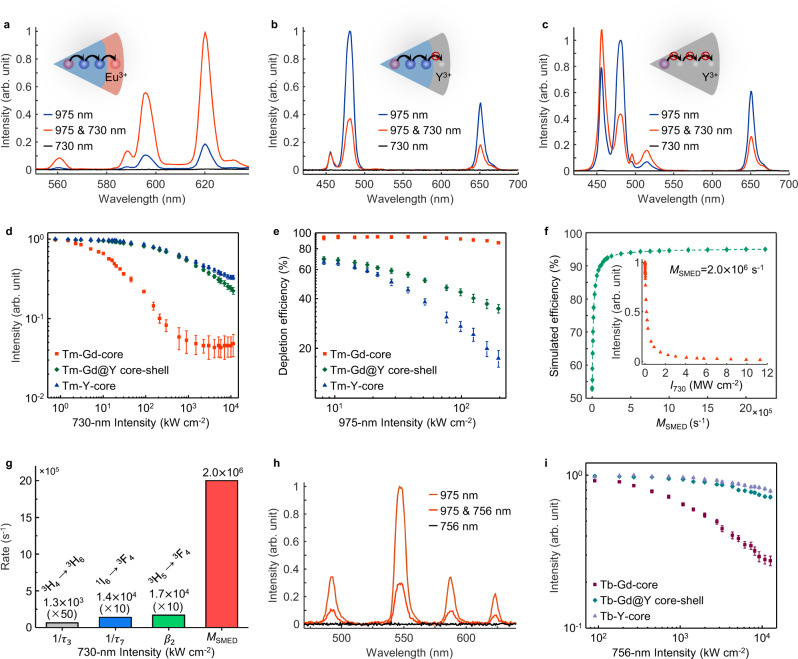
Spectroscopic investigations of SMED using Tm^3+^-activated and Tb^3+^-activated nanoparticles. **a**–**c** Spectra of NaGdF_4_:Yb/Tm (18/0.3%)@NaGdF_4_:Eu (15%), NaGdF_4_:Yb/Tm (18/0.3%)@NaYF_4_, and NaYF_4_:Yb/Tm (18/0.3%) nanoparticles, respectively, co-irradiated with different CW lasers (*I*_975_ = 21.7 kW cm^−2^; *I*_730_ = 3.01 MW cm^−2^). **d**, **e** Comparison of the depletion- or excitation intensity-dependences among NaGdF_4_:Yb/Tm (18/0.3%), NaGdF_4_:Yb/Tm (18/0.3%)@NaYF_4_ and NaYF_4_:Yb/Tm (18/0.3%) nanoparticles (*I*_975_ = 21.7 kW cm^−2^; *I*_730_ = 3.01 MW cm^−2^). **f** Numerical simulation of the relationship between optical depletion of the ^1^G_4_ state and the effect of the SMED mechanism. The inset shows the simulated depletion intensity-dependences when *M*_SEMD_ is 2.0 × 10^6^ s^−1^. **g** Comparison of the energy dissipation rate of SMED with other parameters of the energy decay rate in Tm^3+^-doped systems. 1/*τ*_3_ and 1/*τ*_7_: radiative decay rates of the ^3^H_4_ and ^1^I_6_ states, respectively. *β*_2_: nonradiative decay rate from ^3^H_5_ to ^3^F_4_ states. **h** Depletion efficiency (average at 70%) of multiband emissions from NaGdF_4_:Yb/Tb (12/8%) nanoparticles (*I*_975_ = 67.5 kW cm^−2^; *I*_756_ = 9.20 MW cm^−2^). **i** Comparison of depletion intensity-dependences among NaGdF_4_:Yb/Tb (12/8%), NaGdF_4_:Yb/Tb (12/8%)@NaYF_4_ and NaYF_4_:Yb/Tb (12/8%) nanoparticles with a fixed excitation intensity (*I*_975_ = 67.5 kW cm^−2^). Data in **d**, **e**, **i** were presented as mean value ± SD. Error bars are defined as the SD of *n* = 3 independent measurements.

To validate the critical role of particle surfaces in optical depletion, we further prepared a series of lanthanide-doped nanoparticles with various doping concentrations, compositions, and core-shell structures (Supplementary Table 1). For example, we designed NaGdF_4_:Yb/Tm (18/0.3%)@NaGdF_4_:Eu (15%) core-shell nanoparticles with which specific pathways of energy migration could be activated under two-beam excitation. Spectroscopic data showed that the intensity of Eu^3+^ emission increased by about 6-fold upon 730-nm excitation (Fig. 3a and Supplementary Fig. 3). Since Eu^3+^ emission could not be generated directly with a 730-nm laser, this observed enhancement was attributed to Gd-mediated distant energy transfer from Tm^3+^ to Eu^3+^ across the core-shell interface^28,33^. We also examined the suitability of NaGdF_4_:Yb/Tm (18/0.3%)@NaYF_4_ nanoparticles for SMED microscopy. Under the same conditions, we found a 33% decrease in depletion efficiency for these nanoparticles with inert-shell passivation (Fig. 3b). An increase in the inert-shell thickness further reduced the depletion efficiency (Supplementary Fig. 4). For NaYF_4_:Yb/Tm (18/0.3%) nanoparticles, we recorded a 41% decrease in depletion efficiency (Fig. 3c). This result suggests that the depletion of excitation energy at higher-lying ^1^I_6_ states of Tm^3+^ cannot be efficiently achieved without Gd^3+^-mediated energy migration. With decreasing Gd^3+^ concentration in the Y^3+^-based host lattice, a gradual decrease in depletion efficiency was recorded (Supplementary Fig. 5).

The laser intensity-dependent depletion efficiency further confirms the unique ability of surface quenching to achieve high-efficiency optical depletion. Unlike the fast exponential decay characteristic of NaGdF_4_:Yb/Tm nanoparticles, nanoparticles coated with a thin NaYF_4_ shell exhibited much slower decay with a 67-fold increase in depletion saturation intensity (~1.24 MW cm^−2^) (Fig. 3d). As the ESA process governs optical depletion in yttrium host lattices, NaYF_4_:Yb/Tm nanoparticles displayed a high saturation intensity of 1.55 MW cm^−2^. Notably, the depletion saturation intensity of Y-based nanoparticles is 84 times higher than that of Gd-based nanoparticles. Apart from the depletion intensity dependence, we also measured depletion efficiencies of NaGdF_4_:Yb/Tm nanoparticles and control samples at various excitation intensities (Fig. 3e). When excitation intensity increased from 8.2 to 196 kW cm^−2^, the depletion efficiency of NaGdF_4_:Yb/Tm nanoparticles decreased by only 8% (from 95% to 87%). This feature is particularly attractive because it enables super-resolution microscopy over a wide range of excitation intensities without the need to adjust the depletion intensity. In comparison, the depletion efficiency of NaGdF_4_:Yb/Tm@NaYF_4_ and NaYF_4_:Yb/Tm nanoparticles decreased by 35% and 49%, respectively, with increasing excitation intensity. For NaGdF_4_:Yb/Tm nanoparticles, variations in nanoparticle size have little effect on depletion efficiency (Supplementary Fig. 5).

We next performed rate equation modeling and numerical simulation to probe the photophysical dynamics of Tm-doped NaGdF_4_ materials (see Methods, Supplementary Fig. 6, Supplementary Table 2). The effect of SMED can be treated as a fast nonradiative decay from the ^1^I_6_ state of Tm^3+^ to the ground state, the rate of which is defined as the integrated energy dissipation rate (*M*_SMED_). *M*_SMED_ is proportional to the energy transfer efficiency between Tm^3+^ and Gd^3+^, the migration efficiency within the gadolinium sublattice, and the population of surface quenchers. Depletion efficiency increased sharply with increasing energy dissipation rate, in good agreement with experimental observations (Fig. 3f). According to our modeling, a depletion efficiency of 95% requires a minimum energy dissipation rate of 2.0 × 10^6^ s^−1^, which is two orders of magnitude faster than the nonradiative decay of the ^3^H_5_ → ^3^F_4_ multiphonon relaxation (1.7 × 10^4^ s^−1^)^34^ and three orders of magnitude faster than the intense 800-nm upconversion emission (^3^H_4_ → ^3^H_6_, 1.3 × 10^3^ s^−1^) in conventional Tm^3+^ upconversion systems (Fig. 3g)^34,35^. The fast emission decay through SMED with a rate constant of ~2.0 × 10^6^ s^−1^ leads to largely de-populated ^1^I_6_ and ^1^G_4_ states of Tm^3+^. An analytical solution based on a simplified four-level system further confirms that surface quenchers can quickly dissipate emission energy and substantially lower the depletion saturation intensity (see Methods, Supplementary Fig. 7). To further probe the underlying mechanism of SMED by surface quenchers, we studied the electronic structures of Gd^3+^-doped NaYF_4_ nanocrystals containing various types of defects by performing first-principles calculations based on density functional theory (DFT)^36^ (see Methods, Supplementary Fig. 8). Calculations at the single-particle level suggest that dipole-allowed electronic transitions occur at lattice defects (e.g. fluorine vacancy, oxygen substitution and hydroxyl substitution) due to Förster resonance energy transfer from excited Gd^3+^ ions. Although adsorption of OH^−^, H_2_O, or organic molecules on nanoparticle surfaces can lead to midgap states, the energy differences between these states and host states do not match the ^3^S_7/2_−^6^P_7/2_ gap of Gd^3+^. The adsorbates likely deactivate excited Gd ions by vibronic coupling rather than by Förster resonance energy transfer (Supplementary Fig. 8).

We also observed the same effect of SMED in Tb^3+^-activated nanoparticles^37^. Upon 756-nm beam illumination, the emission bands of Tb^3+^ at 490, 547, 585, and 620 nm generated by a 975-nm excitation beam were suppressed by 70% (Fig. 3h). This depletion was likely due to the electrons in the ^5^D_4_ state of Tb^3+^ being first stimulated to higher-lying states through an ESA process, then transferred to the Gd sublattice, and eventually dissipated at particle surfaces (Supplementary Fig. 3). Similarly, lowered depletion efficiencies (26% or 17%, respectively) were recorded after coating Tb^3+^-activated nanoparticles with an inert-shell or replacing the host material with NaYF_4_ (Fig. 3i).

### SMED enables low-power, high-resolution microscopy

**Fig. 4:**
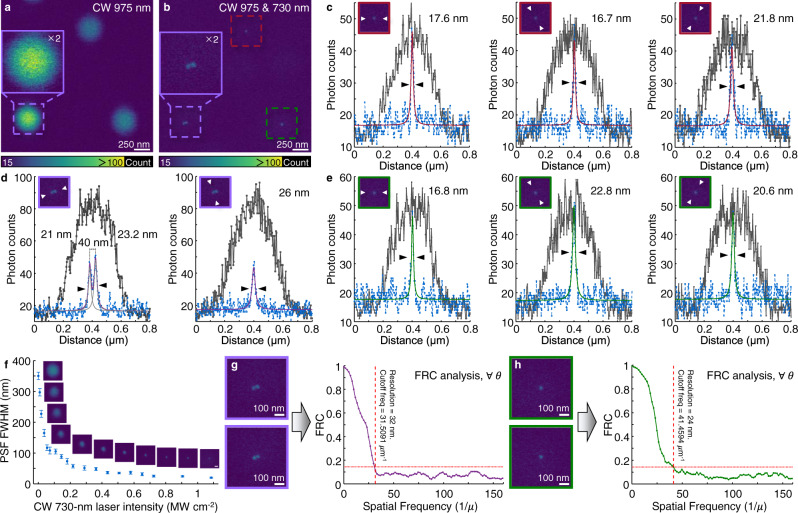
SMED microscopy for single-particle super-resolution imaging. **a**, **b**, Imaging of NaGdF_4_:Yb/Tm (18/0.3%) nanoparticles (~ 11 nm in diameter) with a laser scanning microscope irradiated with a CW 975-nm Gaussian beam (*I*_975_ = 98 kW cm^−2^) and a dual-beam system (975-nm Gaussian beam and CW 730-nm doughnut-shaped beam, *I*_730_ = 1.09 MW cm^−2^, *P*_730_ = 4.85 mW), respectively. Images shown are averages of two repeated. Image dimensions: 800 × 800 pixels; pixel size: 3 nm; pixel dwell time: 200 μs; acquisition time: 130 s. Image reproducibility can be seen in Supplementary Fig. 10. **c**–**e** Intensity profiles of the nanoparticles from red, purple, and green dotted boxes in **a**, **b**, respectively. The intensity profiles were measured radially and the insets show the corresponding measurement directions. **f** Measured effective full-width-at-half-maximum (FWHM) dimension of the point spread function (PSF) versus the depletion power. The measured nanoparticle was marked with green dotted boxes in **a**, **b**. Data were presented as mean value ± SD. Error bars are defined as the SD of *n* = 3 independent measurements. Scale bar: 100 nm. **g**, **h** Image resolution of the nanoparticles from purple and green dotted boxes in **b** determined by Fourier ring correlation (FRC). The signal-to-noise ratio (SNR) of these single-particle images is approximately 5 with a mean detector noise of 10 photon counts. Three pairs of images were used for FRC calculation with similar results obtained.

Based on the correlation between the imaging spot size and laser intensity given by $d={d}_{0}/\sqrt{1+I/{I}_{{{{{{\rm{sat}}}}}}}}$, where *I* and *I*_sat_ are the depletion laser intensity maxima and the depletion saturation intensity, respectively, a low *I*_sat_ would enable SMED microscopy with a significant improvement in image resolution. To validate this hypothesis, we prepared three sets of Tm^3+^-activated SMED nanoparticles with different diameters (8, 11, and 15 nm) (Supplementary Figs. 5, 9). The size-dependent optical responses of these nanoprobes were characterized by microscopic imaging at the single-nanoparticle level. The brightness of the 8-nm nanoparticles (CW, *I*_975_ = 144 kW cm^−2^) is comparable to that of two-photon-excited Texas Red molecules or small agglomerates illuminated with femtosecond pulses, *I*_830_ = 98 kW cm^−2^ (Supplementary Fig. 9). Indeed, imaging NaGdF_4_:Yb/Tm (18/0.3%) nanoparticles (~11 nm) with SMED shows a significant improvement in spatial resolution (Fig. 4a, b). The point spread function (PSF) of single particles in three different regions was radially measured, revealing an effective PSF with average lateral full-width-at-half-maximum (FWHM) dimension of 20 nm (minimum 16.8 nm) or 1/49 of the excitation wavelength of 975 nm (Fig. 4c–e). Compared with the spot size excited exclusively with a 975-nm beam, PSF was narrower by a factor of 17.5 when the depletion intensity reached 1.09 MW cm^−2^ (Fig. 4f). Meanwhile, we acquired two additional super-resolution images of the same area under the same conditions to reduce random error (Supplementary Fig. 10). Fourier ring correlation (FRC) was also used to determine the resolution of SMED images, yeilding values slightly larger than those from FWHM analysis (about 24–32 nm) (Fig. 4g, h). Furthermore, no re-excitation emission background was detected via SMED, and these nanoparticles exhibited high photostability across more than 5 h of laser scanning and imaging (Supplementary Fig. 11)^16,19,22^. Coordinate-targeted SMED microscopy can be easily performed with a pair of low-power CW lasers without the need for complicated image reconstruction and setup^27,38,39^.

**Fig. 5:**
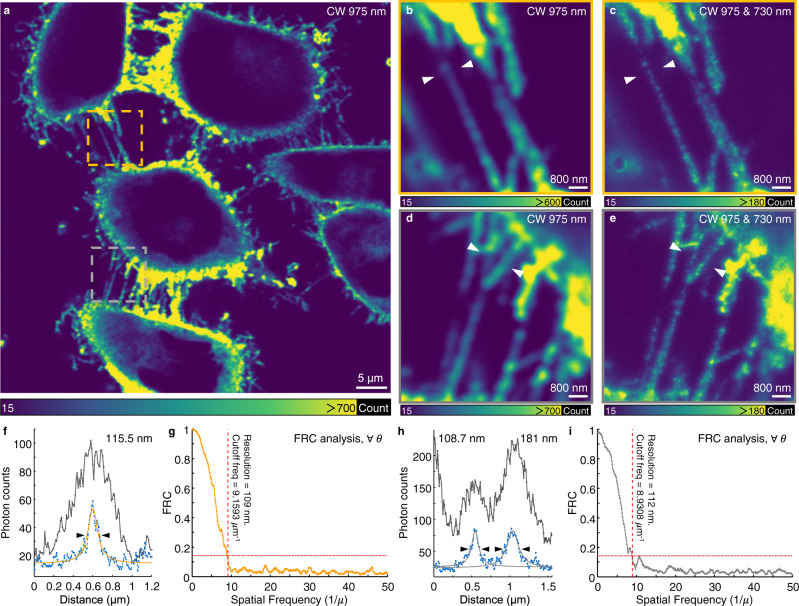
SMED microscopy for super-resolution subcellular imaging. **a** Conventional upconversion imaging of actin filaments in fixed Hela cells stained with phalloidin-functionalized Tm^3+^-activated nanoparticles with a diameter of 8 nm. (image dimensions: 1024 × 1024 pixels; pixel size: 62 nm; pixel dwell time: 200 μs; acquisition time: 212 s; *I*_975_ = 98 kW cm^−2^, *I*_730_ = 537 kW cm^−2^, *P*_730_ = 2.4 mW. More than three independent labeling and imaging experiments showed similar results.) **b**–**e** Conventional- and super-resolution images of selected areas from yellow (**b**, **c**) and gray (**d**, **e**) dotted boxes marked in **a**. Images shown are averages of two repeated. Image dimensions: 800 × 800 pixels; pixel size: 10 nm; pixel dwell time: 200 μs; acquisition time: 130 s. **f** Corresponding intensity profiles of the features marked in **b**, **c**. **g** The resolution of image **c** determined by FRC. **h** Corresponding intensity profiles of the features marked in **d**, **e**. **i** Resolution of the image **e** determined by FRC. Intensity profiles were fitted using the Lorentzian function and the resolution value was determined by the FWHM of the Lorentzian curves. The criterion of 0.143 was used in the FRC analysis.

We next performed surface bioconjugation to 8-nm Tm^3+^-activated nanoparticles using actin-targeted phalloidin and specifically labeled them on actin filaments of fixed HeLa cells for super-resolution imaging (Fig. 5a–e and Supplementary Figs. 12, 13). Fluorescence and bright-field imaging of the cells showed high staining efficiency and significant improvement in resolution (with a lateral effective FWHM of 108 nm and a FRC resolution of 109 nm) (Fig. 5f–i and Supplementary Fig. 13). Limited by the sizes of filaments observed and the state-of-the-art of subcellular labeling with lanthanide-doped nanoprobes, the achievable imaging resolution in biological environments differs from that of single-particle imaging experiments. With further developments in bioconjugation^40^, SMED microscopy may allow observation of biological structures down to the imaging resolution limit of 8–20 nm^41,42^.

## Discussion

The 95% depletion efficiency we achieved in NaGdF_4_:Yb/Tm nanoparticles under 730-nm irradiation with a low depletion saturation intensity of 18.3 kW cm^−2^ contrasts starkly with imaging conditions typically employed in conventional STED microscopy. The low stimulated emission cross-sections of lanthanide-doped nanoparticles can be compensated through gadolinium lattice-mediated surface energy migration. The work presented here provides insight into investigations of other nanoparticle systems in which spatial confinement of energy migration can be well controlled^43^. Given the tunable nature of upconversion emission color and lifetime, NaGdF_4_-based SMED offers unparalleled multiplexing and multicolor imaging capabilities. Out-of-focus upconversion emission can be easily suppressed in SMED imaging by employing confocal detection, thereby enabling high-resolution, three-dimensional optical sectioning in biological samples. Our results highlight the potential advantages of surface-mediated energy migration as a powerful tool that complements previously reported STED/RESOLFT imaging techniques (Supplementary Table 3). A more comprehensive understanding of the SMED mechanism described here and precise control over nanoparticle surface states may enable further development of high-performance luminescent probes for wide-ranging biophotonic and bioimaging applications. The SMED strategy, therefore, provides a facile route to achieve low-power, diffraction-unlimited photoactivation^44^, optical data storage^45^, and lithography^46^.

## Methods

### Materials

Thulium(III) acetate hydrate (99.9%), gadolinium(III) acetate hydrate (99.9%), ytterbium(III) acetate hydrate (99.9%), yttrium(III) acetate hydrate (99.9%), terbium(III) acetate hydrate (99.9%), europium acetate hydrate (99.9%), diethylene glycol (DEG, 98%), Poly-L-lysine solution (0.1 wt%), *N*-(3-dimethylaminopropyl)-*Nʹ*-ethylcarbodiimide hydrochloride (EDC, ≥ 98%) and *N*-hydroxysuccinimide (NHS, 98%) were purchased from Sigma-Aldrich. Sodium hydroxide (NaOH, ≥98%), ammonium fluoride (NH_4_F, ≥ 99.99%), 1-octadecene (ODE, ≥ 90%(GC)), oleic acid (OA, AR), cyclohexane (AR, 99.5%) and ammonia solution (NH_3_·H_2_O, 25–28%) were purchased from Aladdin®, China. Methanol (AR), ethanol (AR), ether (AR), hydrochloric acid (HCl, AR, 36–38%) and dimethylsulfoxide (DMSO, AR) were purchased from Sinopharm Chemical Reagent Co., China. Poly(acrylic acid) (PAA, M.W~2000) was purchased from Shanghai Macklin Biochemical Co., Ltd. Compound 2-(*N*-morpholino)ethanesulfonic acid (MES, 0.1 M, pH = 6.0) was purchased from Leagene Biotechnology co., Beijing. Phosphate-buffered Saline (PBS), ProLong™ Gold Antifade Mountant, Alexa Fluor™ Plus 405 Phalloidin (A30104, Invitrogen™), organic fluorophore Texas Red (>90% single isomer, T6256, Invitrogen™) were purchased from Thermo Fisher Scientific. Amino-phalloidin was purchased from Shanghai Maokang Biotechnology Co. Ltd., China. Paraformaldehyde (PFA, 4%) was purchased from Invitrogen. Triton X-100 was purchased from Amresco. Quick Block blocking buffer, bovine serum albumin (BSA, ≥ 99%, fatty acid & IgG free, BioPremium) and ethanesulfonic acid (HEPES) buffer (0.01 M, pH = 7.3) were purchased from Beyotime, China. HeLa cells source was provided by Sun Yat-Sen University (Guangzhou, China). All the reagents were used as received without any further purification.

### Synthesis of 11-nm NaGdF_4_:Yb/Tm nanoparticles

Lanthanide-doped nanoparticles were synthesized using previously reported protocols with modifications^47–49^. To a 100-mL round-bottom flask, a mixture of 10 mL OA and 15 mL ODE was added, followed by addition of a 5-mL stock solution of Ln(CH_3_CO_2_)_3_ (Ln = Gd/Yb/Tm) (0.2 M). The mixture was heated to 150 °C and kept for 40 min under stirring to form a lanthanide-oleate precursor solution, which was slowly cooled to room temperature before a NaOH-methanol stock solution (1 M, 2.5 mL) and an NH_4_F-methanol stock solution (0.4 M, 10 mL) were pipetted into the flask. Next, the mixture was kept at 40 °C under stirring for an additional 2 h. Subsequently, the solution was heated to 100 °C for 30 min under a vacuum to evaporate the methanol. After removing methanol, the solution temperature was further elevated and kept at 300 °C under stirring for 1.5 h in an argon atmosphere for nanoparticle growth. After the reaction, the solution was cooled to room temperature naturally, precipitated by adding 15-mL ethanol, and then centrifugated at 7500 r.p.m. (relative centrifuge force (RCF) = 5840 × g) for 5 min. The obtained NaGdF_4_:Yb/Tm nanoparticles were washed with ethanol and cyclohexane three times and then re-dispersed in 8-mL cyclohexane. NaGdF_4_:Yb/Tb nanoparticles were synthesized using the same protocol. Note that the mixture volumes of OA and ODE were adjusted to 7.5 and 17.5 mL, respectively, for the NaYF_4_ host matrix.

### Synthesis of 8-nm NaGdF_4_:Yb/Tm nanoparticles

The synthetic procedure of 8-nm NaGdF_4_:Yb/Tm is similar to that of 11-nm NaGdF_4_:Yb/Tm nanoparticles. While keeping the volumes and ratio of Ln(CH_3_CO_2_)_3_ unchanged, the volumes of OA and ODE were changed to 7 and 15 mL. In addition, the reaction temperature was adjusted from 300 to 260 °C, and the reaction time shortened from 1.5 to 1 h.

### Synthesis of 15-nm NaGdF_4_:Yb/Tm nanoparticles

NaGdF_4_:Yb/Tm nanoparticles of 15 nm in diameter were obtained by growing the same layer onto the as-prepared 11-nm core NaGdF_4_:Yb/Tm nanoparticles^10^. First, a stock solution of 10-mL OA, 15-mL ODE and 5-mL Ln(CH_3_CO_2_)_3_ (Ln = Gd/Yb/Tm) (0.2 M) was added into a 100-mL round-bottom flask. The reaction solution was heated to 150 °C and kept for 40 min under stirring to form a clear solution, and then cooled to 80 °C. An 8-mL suspension of the as-prepared 11-nm core NaGdF_4_:Yb/Tm nanoparticles was pipetted into the flask (the core-to-shell volume ratio, 1:1) and maintained at this temperature for 30 min to remove cyclohexane. Subsequently, the solution was cooled slowly to room temperature, and then 2.5-mL NaOH-methanol stock solution (1.0 M) and 10 mL NH_4_F-methanol stock solution (0.4 M) were pipetted into the flask. The following steps were the same as those used to synthesize bare core 11-nm NaGdF_4_:Yb/Tm nanoparticles. The obtained 15-nm NaGdF_4_:Yb/Tm nanoparticles were re-dispersed in 8-mL cyclohexane.

### Synthesis of 17-nm NaGdF_4_:Yb/Tm@NaYF_4_ core-shell nanoparticles

To epitaxially grow an inert NaYF_4_ shell, 7.5-mL OA, 17.5-mL ODE and 5-mL Y(CH_3_CO_2_)_3_ stock solution (0.2 M) were added into a 100-mL round-bottom flask containing 15-nm NaGdF_4_:Yb/Tm nanoparticles. The core-to-shell volume ratio is 1:1. The obtained 17-nm NaGdF_4_:Yb/Tm@NaYF_4_ nanoparticles were re-dispersed in 8-mL cyclohexane.

### Synthesis of 28-nm NaGdF_4_:Yb/Tm@NaYF_4_ core-shell nanoparticles

The 28-nm NaGdF_4_:Yb/Tm@NaYF_4_ core-shell nanoparticles were obtained using the same procedure as 17-nm NaGdF_4_:Yb/Tm@NaYF_4_ core-shell nanoparticles, except for the change in the core-to-shell volume ratio from 1:1 to 1:4. A 2-mL suspension of the as-prepared 11-nm NaGdF_4_:Yb/Tm nanoparticles was added. The obtained 28-nm NaGdF_4_:Yb/Tm@NaYF_4_ nanoparticles were re-dispersed in 4-mL cyclohexane.

### Synthesis of 38-nm NaGdF_4_:Yb/Tm@NaYF_4_ nanoparticles

To further increase NaYF_4_ shell layer thickness, another NaYF_4_ layer was epitaxially grown onto the as-prepared 28-nm NaGdF_4_:Yb/Tm@NaYF_4_ nanoparticles using the standard core-shell synthesis. The core-to-shell volume ratio is 1:2, with the addition of a 4-mL suspension of 28-nm NaGdF_4_:Yb/Tm@NaYF_4_ nanoparticles. The obtained 38-nm NaGdF_4_:Yb/Tm@NaYF_4_ nanoparticles were re-dispersed in 4-mL cyclohexane.

### Preparation of nanoparticle-coated slides for spectroscopic study

To perform spectroscopic and emission depletion characterizations, the sample cover slides with well-dispersed nanoparticles (10.0 μL, 200.0 μg/mL) were prepared by spin-coating (6000 r.p.m, RCF = 3740 × *g*). The employed aqueous sample was transferred directly from hydrophobic cyclohexane before spin-coating according to a previously reported hydrochloric acid treatment^50^. After air-drying, the sample coverglass was placed on a clean glass slide spread with a small drop of embedding medium (ProLong™ Gold Antifade Mountant, Invitrogen) to ensure refractive index matching. Air bubbles were gently extruded and the fringe was properly sealed with nail polish. The sample was kept at room temperature and in the dark for another 10 h to ensure complete drying before measurement.

### Sample preparation for single-nanoparticle imaging

To perform single-nanoparticle imaging, sample slides coated with lanthanide-doped nanoparticles were prepared according to literature methods^10,24^. To prepare a sample slide, a high precision microscope cover glass (thickness No.1.5H, 170 ± 5 μm, Deckgläser, Paul Marienfeld GmbH) was washed with anhydrous ethanol/ultrapure water under ultrasonication and then treated with 60-μL poly-L-lysine solution (0.1% in H_2_O w/v). After about half an hour, the poly-L-lysine was washed off with ultrapure water, and the cover glass was air-dried. A 20.0-μL nanoparticle solution (0.1 μg/mL in cyclohexane) was added to the treated surface, immediately followed by wash three times with cyclohexane. After air-drying, the cover glass was placed on a clean glass slide spread with a small drop of embedding medium. Air bubbles were extruded and the glasses were sealed with nail polish. The sample was kept at room temperature for another 10 h to ensure complete drying before measurement.

### Preparation of gold nanoparticle sample slides

To measure the PSF of the lasers, sample slides with dispersed gold nanoparticles (CTAB-capped, 120 nm in diameter, NS-120-50, NanoSeedz) were prepared. A 20.0-μL water solution of gold nanoparticle (ten times diluted from the original solution) was directly spin-coated onto a cover slide. After air-drying, the cover slide was placed on a clean glass slide spread with a small drop of embedding medium (ProLong™ Gold Antifade Mountant, Invitrogen). Air bubbles were extruded and the glasses were sealed with nail polish. The sample was kept at room temperature for another 10 h to ensure complete drying before measurement.

### Preparation of Texas Red-stained slides

According to a literature method^51^, the sample for single-molecule two-photon microscopic imaging was prepared by spin coating of a DMSO solution of Texas Red molecules (20.0 μL, 2 × 10^−7^ M) at 6000 r.p.m (RCF = 3740 × *g*) onto a cover slide. After air-drying, the cover glass was placed on a clean glass slide spread with a small drop of embedding medium (ProLong Gold Antifade Mountant) to relieve the photobleaching of Texas Red. Air bubbles were also avoided and the glasses were sealed with nail polish. The sample was kept at room temperature and in the dark for another 10 h to ensure complete dryness before measurement.

### Preparation of PAA-coated lanthanide-doped nanoparticles

PAA-coated nanoparticles of 8-nm in diameter for biomarkers were prepared using a general ligand exchange method^52^, and the procedures are similar to our previous work^25^. To a 100-mL round-bottom flask, 20 mL of DEG and 0.2 g of PAA were added. The mixture was heated to 110 °C and kept at this temperature for 1 h under an argon atmosphere. Next, 2 mL of OA-nanoparticles were injected into the solution and maintained at the same temperature for 30 min under vacuum to evaporate cyclohexane. When the cyclohexane was evaporated, the solution was heated to 240 °C and kept at this temperature for 3 h under an argon atmosphere. After the solution was cooled to room temperature naturally, 15 mL of 1% HCL were added and the solution was continued to stir for 20 min. The obtained PAA-coated nanoparticles were precipitated by centrifugation for 30 min (18,000 r.p.m (RCF = 33,660 × *g*)), then washed with ethanol and ultrapure water (*v*/*v* = 1:1) three times, and re-dispersed in 2-mL ultrapure water.

### Activation of the carboxyl groups of PAA-coated lanthanide-doped nanoparticles

Following the commonly used methods^53,54^, the protocol is similar to our previous work^25^. Firstly, EDC and NHS solids were dissolved in MES solution at a concentration of 0.2 and 0.3 mg μL^−1^, respectively. Then 1 mL of PAA-coated nanoparticle solution (1 mg mL^−1^), 20 μL of EDC and 20 μL of NHS were added into a 1.5-mL centrifuge tube and sonicated for 30 s. The mixture was stirred for 2 h at room temperature, then centrifugated for 30 min to remove the redundant EDC and NHS. (18,000 r.p.m (RCF = 33,660 × *g*)). The precipitate was collected and re-dispersed in 1 mL ultrapure water. The pH value of the solution was adjusted to 7.2–7.5 by adding an ammonia solution (2 M).

For preparing the Phalloidin conjugated 8-nm nanoparticles^55^, 10 μL amino-Phalloidin DMSO stock solution (100 μM) and 110 μL HEPES buffer were added to 30 μL activated PAA-lanthanide nanoparticles (1.0 mg mL^−1^). The mixture was stored in a refrigerator at 4 °C for another 4 h. Followed by centrifugation at 18,000 r.p.m (RCF = 33,660 × *g*). for 20 min to remove the excess of amino-Phalloidin, the obtained precipitate was re-dispersed into 150 μL HEPES buffer.

### Labeling of cytoskeleton actin filaments using lanthanide-doped nanoparticles

These procedures followed the commonly used cell labeling method^19,53^, similar to our previous work^25^. The HeLa cells were cultured in 96-well glass-bottom plates (thickness 170 ± 5 μm, P96-1.5H-N, Cellvis) with 15,000 cells in each unit, with the temperature set at 37 °C, the humidity at 95%, and the CO_2_ concentration at 5% overnight (>12 h). The cells were rinsed three times using 200-μL pre-warmed PBS buffer (37 °C, 5 min each) to remove the residual culture media and then fixed using PFA for 15 min at room temperature. Subsequently, the cells were rinsed three times using PBS buffer to remove the residual PFA. Next, the cells were permeabilized in 200-μL 0.2% Triton X-100 for 30 min at room temperature and rinsed three times using HEPES buffer. 200 μL Quick Block blocking buffer was added to block the fixed cells for 20 min at room temperature. When the procedure of blocking was completed, 150 μL of freshly prepared phalloidin-bioconjugated lanthanide-doped nanoparticles (with 1% bovine serum albumin in buffer (200 μg mL^−1^) were added. The cells were kept at room temperature for 4 h and then rinsed twice with pre-warmed (37 °C) HEPES buffer. Finally, the HEPES buffer was removed and a small drop of embedding medium was dropped into the well. The sample was kept at room temperature and in the dark for 24 h to ensure complete dryness before measurement.

### Labeling of cytoskeleton actin filament using Alexa Fluor Plus 405 Phalloidin

To prepare the stock solution of Alexa Fluor Plus 405 Phalloidin, the contents of the vial were dissolved in 150 μL of anhydrous DMSO to yield a 300 × stock solution, which is equivalent to approximately 66 μM. The staining solution was prepared by diluting 0.5 μL of the 300 × stock solution into 150 μL of PBS and adding 1% BSA. When fixation, permeabilization, and blocking were completed, 150 μL of the freshly prepared staining solution was added. The cells were kept at room temperature for 4 h and then rinsed twice with pre-warmed (37 °C) PBS buffer.

### Transmission electron microscopy (TEM)

The size and morphology of as-prepared nanoparticles were obtained using a transmission electron microscope JEM-2100HR, JEOL (200 kV). The bioconjugated lanthanide-doped nanoprobes were diluted and dispersed in ultrapure water, whereas other samples were diluted and dispersed in cyclohexane, then dropped onto the surface of copper grids for the test of TEM.

### Optical system for spectroscopic study and emission depletion measurements

A lab-made optical system coupled with two laser beams was built to implement spectroscopic measurement, luminescence lifetime measurement, and super-resolution imaging. As the optical system’s layout shown in Supplementary Fig. 1, for the spectroscopic study of Tm^3+^-activated nanoparticles, the excitation 975-nm CW laser beam was generated by a single-mode diode laser (Laser 1, B&A Technology Co. Ltd.). The depletion 730-nm CW laser beam was generated by a Ti:sapphire laser (Laser 2, Mira 900, Coherent). Two band-pass filters (F1, LD01-975/10-12.5, Semrock; F2, FF01-730/39-25, Semrock) were used to clean the spectrum of the two lasers. Two pairs of half-wave plates and polarization beam splitters were used to control the laser beam power. In the optical excitation pathway, the excitation laser beam was firstly expanded by a pair of lenses with 25-mm and 100-mm focus lengths (L1, L2), then optimized by a spatial filter which consisted of a pair of 50-mm focus length lenses (L3, L4) and a 25-μm gold-coated pinhole (PH1). Half-wave plate (HWP2) and quarter-wave plate (QWP1) were used to further regulate the profile of excitation beam. In the optical depletion pathway, the depletion laser beam was firstly optimized by a spatial filter which consisted of a pair of 50-mm focus length lenses (L5, L6) and a 25-μm gold-coated pinhole (PH2), then optimized and shrank by another spatial filter which consisted of a pair of lenses with 50-mm and 25-mm focus lengths (L7, L8) and a 50-μm gold-coated pinhole (PH3). Two diaphragms (D2, D3) were placed in front and behind the second spatial filter, respectively. Half-wave plate (HWP5) and quarter-wave plate (QWP2) were used to further regulate the profile of depletion beam. The positions of lenses L4 and L8 were controlled with three-dimensional displacement stages. During the spectroscopic study, the sizes of Gaussian excitation beam and Gaussian depletion beam were modulated to be the same. The two laser beams were spatially superimposed using a 950-nm short-pass dichroic mirror (DM1, ZT950spxrxt, Chroma), and multiple mirrors in the optical path were used to calibrate the optical system. Then the coupled two beams were further filtered by a 715-nm long-pass emission filter (F3, FF01-715/LP-25, Semrock) and then directed into an commercially available multiphoton laser scanning microscope (FV1000MPE-S with motorized inverted IX81, Olympus), where two laser beams were reflected by a 690-nm short-pass dichroic mirror (DM2), then focused on the samples through an oil-immersed objective (OL, 100 × /NA = 1.45, UPLXAPO100XO, Olympus). These two laser beams were carefully aligned to ensure precise 3D overlap of their PSFs in XYZ directions. The upconversion emission was collected by the same objective and also filtered by a 694-nm short-pass emission filter (F4, FF01-694/sp-25, Semrock). A spectrometer (QE65Pro, Ocean Optics) was used to collect the emission spectra. For the spectroscopic study of Tb^3+^-activated nanoparticles, The depletion 756−nm CW laser beam was also generated by a Ti:sapphire laser (Laser 2, Mira 900, Coherent) and the filter was changed accordingly. All measurements were performed under ambient, strict light-shielding conditions.

### Conventional upconversion laser-scanning imaging and super-resolution imaging

For microscopic imaging, the multiphoton laser scanning microscope (FV1000MPE-S with motorized inverted IX81, Olympus) was configurated with both Galvanometer scanning mirrors unit and non-descanned detection (NDD) module with a high-sensitivity photomultiplier tube (PMT1) employed as a single-photon counting detector for fluorescence imaging (Supplementary Fig. 1). In addition, the system was also configured with differential interference contrast (DIC) components for transmission bright field imaging (PMT2), and a single 730 nm beam was used to perform DIC bright-field cell imaging. For laser scanning imaging of Tm^3+^-activated nanoparticles, in front of the PMT1, a 720-nm short-pass filter (F5, FF01-720/SP-25, Semrock), a 665-nm short-pass filter (F6, FF01-665/SP-25, Semrock), a 458-nm long-pass dichroic mirror (DM3, ZT458rdc, Chroma) and a 480-nm band-pass filter (F7, ET480/20x, Chroma) were used to block the laser light and yield the 475-nm emission of interest. To implement super-resolution microscopy, a 730-nm vortex phase plate (VR1-730-SP, LBTEK) was placed in the depletion optical path to generate the doughnut-shaped point spread function (PSF) for 730 nm beam. A diaphragm (D1) was placed in front of the VPP. HWP5 and QWP2 were precisely modulated to regulate the polarization state for a high-quality zero center. The Gaussian excitation beam and doughnut-shaped depletion beam were aligned to ensure precise 3D overlap of their PSFs in XYZ directions.

For imaging the scattered/reflected light from gold nanoparticles, the 690-nm short-pass dichroic mirror (DM2) was replaced with a 950-nm short-pass dichroic mirror (ZT950spxrxt, Chroma) for the 975-nm excitation beam or a 700-nm short-pass dichroic mirror (T700spxr-UF3, Chroma) for the 730-nm depletion beam, respectively. Filters F4, F5, F6, F7 and dichroic mirror DM3 were removed, instead, a 715-nm long-pass emission filter (FF01-715/LP-25, Semrock) was placed in front of the PMT1. For the two-photon excitation microscopic imaging of Texas Red, the excitation 830-nm femtosecond pulsed laser was generated by the mode-locked Ti:sapphire laser (Laser 2, Mira 900, Coherent). Filters F6, F7, and dichroic mirror DM3 were removed to pass the emission spectrum (590–690 nm) of Texas Red. For the two-photon excitation microscopic imaging of fixed Hela cells stained with Alexa Fluor Plus 405 Phalloidin, the femtosecond pulsed wavelength was shifted to 850 nm. Filters F6, F7, and dichroic mirror DM3 were also removed accordingly.

### Luminescence lifetime measurement

To measure the emission lifetime of lanthanide-doped nanoparticles, an optical chopper (SR540, Stanford Research Systems, Inc.) was used to modulate the 975-nm excitation beam (Supplementary Fig. 1). The trigger signal from the chopper and the detected photon counts by the PMT1 were synchronized with a time-correlated single-photon counting (TCSPC) system (NanoHarp 200, PicoQuant). The modulation frequency was set to be 1 kHz for the measurements. To minimize the influence of the instrument response function, for example, the falling edge of the mechanically modulated light pulse, the beam was suitably focused (Supplementary Fig. 1) and the chopper was placed in the focal plane of L1. In addition, the instrument response function was considered and measured with a Texas Red ensemble sample with a lifetime on the order of nanoseconds.

### Power density determination

An optical power meter (PM100D, Thorlabs) with microscope slide power meter sensor head (S170C, Si, Thorlabs) was used to determine the laser power. The power output from the objective lens was directly measured by attaching the sensor head to the front of the oil-immersed objective lens. During the experiment, the depletion power was recorded by the power meter by using an uncoated pellicle beam splitter to reflect a small fraction of the laser beam (~5%). The ratio of the laser powers measured outside the microscope and that output from objective lens were calculated to determine the power during imaging or spectroscopy experiments. To calculate the power density of Gaussian beam, the diameter of the laser focus spot was defined as the full width at 1/e^2^ (13.5%) of maximum, and the laser intensity was calculated by dividing the power at objective lens by the area of laser spot. Similar to the calculation of Gaussian beam, the area where the intensity exceeds 1/e^2^ (13.5%) of the maximum was considered as the effective area of doughnut-shaped beam.

### Numerical simulation of the emission depletion in NaGdF_4_:Yb/Tm with SMED mechanism

According to the energy transfer processes in Yb^3+^/Tm^3+^-codoped system, the rate equation set of each energy state can be used to describe the dual-beams excitation/depletion optical process. Theoretical modeling can be developed to elucidate the proposed SMED depletion mechanism in our work. The corresponding energy diagram is shown in Supplementary Fig. 6.

1 $${{{{{{\rm{Tm}}}}}}}^{3+}\Big({}^{3}{{{{{\rm{H}}}}}}_{6}\Big):\frac{d{n}_{0}}{dt}=-{\sum }_{i=1}^{7}\frac{d{n}_{i}}{dt}$$

2 $${{{{{{\rm{Tm}}}}}}}^{3+}\Big({}^{3}{{{{{\rm{F}}}}}}_{4}\Big) : \frac{d{n}_{1}}{dt}={\beta }_{2}{n}_{2}+{c}_{1}{n}_{5}{n}_{3}+{c}_{2}{n}_{3}{n}_{5}+2{c}_{3}{n}_{3}{n}_{0} \\+{b}_{51}\frac{{n}_{5}}{{\tau }_{5}}+{b}_{61}\frac{{n}_{6}}{{\tau }_{6}}+{b}_{71}\frac{{n}_{7}}{{\tau }_{7}}-{w}_{2}{n}_{{{{{{\rm{Yb}}}}}}1}{n}_{1}-\frac{{n}_{1}}{{\tau }_{1}}$$

3 $${{{{{{\rm{Tm}}}}}}}^{3+}\Big({}^{3}{{{{{\rm{H}}}}}}_{5}\Big):\frac{d{n}_{2}}{dt}={\beta }_{3}{n}_{3}+{w}_{1}{n}_{{{{{{\rm{Yb}}}}}}1}{n}_{0}+{b}_{52}\frac{{n}_{5}}{{\tau }_{5}}+{b}_{62}\frac{{n}_{6}}{{\tau }_{6}}-{\beta }_{2}{n}_{2}$$

4 $${{{{{{\rm{Tm}}}}}}}^{3+}\Big({}^{3}{{{{{\rm{H}}}}}}_{4}\Big):\frac{d{n}_{3}}{dt}={\beta }_{4}{n}_{4}+{b}_{73}\frac{{n}_{7}}{{\tau }_{7}}+{c}_{4}{n}_{6}{n}_{0}-{c}_{1}{n}_{5}{n}_{3}-{c}_{2}{n}_{3}{n}_{5}\\ - {c}_{3}{n}_{3}{n}_{0}-{w}_{3}{n}_{{{{{{\rm{Yb}}}}}}1}{n}_{3}-{\beta }_{3}{n}_{3}-\frac{{n}_{3}}{{\tau }_{3}}$$

5 $${{{{{{\rm{Tm}}}}}}}^{3+}\Big({}^{3}{{{{{\rm{F}}}}}}_{3}/{}^{3}{{{{{\rm{F}}}}}}_{2}\Big):\frac{d{n}_{4}}{dt}={\beta }_{5}{n}_{5}+{b}_{74}\frac{{n}_{7}}{{\tau }_{7}}+{c}_{4}{n}_{6}{n}_{0}+{w}_{2}{n}_{{{{{{\rm{Yb}}}}}}1}{n}_{1}-{\beta }_{4}{n}_{4}$$

6 $${{{{{{\rm{Tm}}}}}}}^{3+}\Big({}^{1}{{{{{\rm{G}}}}}}_{4}\Big):\frac{d{n}_{5}}{dt}={\beta }_{6}{n}_{6}+{b}_{75}\frac{{n}_{7}}{{\tau }_{7}}+{w}_{3}{n}_{{{{{{\rm{Yb}}}}}}1}{n}_{3}-{c}_{1}{n}_{5}{n}_{3}\\ - {c}_{2}{n}_{3}{n}_{5}-{\beta }_{5}{n}_{5}-\frac{{n}_{5}}{{\tau }_{5}}+\frac{{\sigma }_{{{{{{\rm{d}}}}}}}^{se}{I}_{{{{{{\rm{d}}}}}}}}{h{v}_{{{{{{\rm{d}}}}}}}}{n}_{7}-\frac{{\sigma }_{{{{{{\rm{d}}}}}}}^{a}{I}_{{{{{{\rm{d}}}}}}}}{h{v}_{{{{{{\rm{d}}}}}}}}{n}_{5}$$

7 $${{{{{{\rm{Tm}}}}}}}^{3+}\Big({}^{1}{{{{{\rm{D}}}}}}_{2}\Big):\frac{d{n}_{6}}{dt}={\beta }_{7}{n}_{7}+{c}_{1}{n}_{5}{n}_{3}+{c}_{2}{n}_{3}{n}_{5}-{c}_{4}{n}_{6}{n}_{0}\\ - {w}_{4}{n}_{{{{{{\rm{Yb}}}}}}1}{n}_{6}-{\beta }_{6}{n}_{6}-\frac{{n}_{6}}{{\tau }_{6}}$$

8 $${{{{{{\rm{Tm}}}}}}}^{3+}\Big({}^{1}{{{{{\rm{I}}}}}}_{6}\Big):\frac{d{n}_{7}}{dt}={w}_{4}{n}_{{{{{{\rm{Yb}}}}}}1}{n}_{6}-{\beta }_{7}{n}_{7}-\frac{{n}_{7}}{{\tau }_{7}}-\frac{{\sigma }_{{{{{{\rm{d}}}}}}}^{se}{I}_{{{{{{\rm{d}}}}}}}}{h{v}_{{{{{{\rm{d}}}}}}}}{n}_{7} \\+\frac{{\sigma }_{{{{{{\rm{d}}}}}}}^{a}{I}_{{{{{{\rm{d}}}}}}}}{h{v}_{{{{{{\rm{d}}}}}}}}{n}_{5}-{k}_{{{{{{\rm{et}}}}}}}{\varphi }_{{{{{{\rm{gd}}}}}}}{n}_{{{{{{\rm{defect}}}}}}}{n}_{7}$$

9 $${{{{{{\rm{Yb}}}}}}}^{3+}\Big({}^{2}{{{{{\rm{F}}}}}}_{7/2}\Big):\frac{d{n}_{{{{{{\rm{Yb}}}}}}0}}{dt}=-\frac{d{n}_{{{{{{\rm{Yb}}}}}}1}}{dt}$$

10 $${{{{{{\rm{Yb}}}}}}}^{3+}\Big({}^{2}{{{{{\rm{F}}}}}}_{5/2}\Big):\frac{d{n}_{{{{{{\rm{Yb}}}}}}1}}{dt}=\frac{{\sigma }_{{{{{{\rm{p}}}}}}}^{a}{I}_{{{{{{\rm{p}}}}}}}}{h{v}_{{{{{{\rm{p}}}}}}}}{n}_{{{{{{\rm{Yb}}}}}}0}-({w}_{1}{n}_{0}+{w}_{2}{n}_{1}+{w}_{3}{n}_{3}+{w}_{4}{n}_{6}){n}_{{{{{{\rm{Yb}}}}}}1}-\frac{{n}_{{{{{{\rm{Yb}}}}}}1}}{{\tau }_{{{{{{\rm{Yb}}}}}}1}}$$

Here, *n*_*i*_ (*i* = 0 to 7) represents the population of Tm^3+^ ions on the ^3^H_6_, ^3^F_4_, ^3^H_5_, ^3^H_4_, ^3^F_3_/^3^F_2_, ^1^G_4_, ^1^D_2_ and ^1^I_6_ states, respectively. *n*_Yb0_ and *n*_Yb1_ represent the population of Yb^3+^ ions on the ^2^F_7/2_ and ^2^F_5/2_ states. *β*_*i*_ (*i* = 2 to 7) represents the non-radiative decay rates of the ^3^H_5_, ^3^H_4_, ^3^F_3_/^3^F_2_, ^1^G_4_, ^1^D_2_ and ^1^I_6_ states of Tm^3+^ ions. *τ*_*i*_ (*i* = 1, 3, 5, 6, 7) represents the radiative lifetimes of the ^3^F_4_, ^3^H_4_, ^1^G_4_, ^1^D_2_ and ^1^I_6_ states of Tm^3+^ ions, respectively, while $\frac{1}{{\tau }_{i}}$ represents the radiative decay rates of the corresponding states. *c*_*i*_ (*i* = 1, 2, 3, 4) denotes the coefficients of cross-relaxation processes, ^1^G_4_ + ^3^H_4_ → ^1^D_2_ + ^3^F_4_, ^3^H_4_ + ^1^G_4_ → ^1^D_2_ + ^3^F_4_, ^3^H_4_ + ^3^H_6_ → ^3^F_4_ + ^3^F_4_ and ^1^D_2_ + ^3^H_6_ → ^3^F_2_ + ^3^H_5_. *w*_*i*_ (*i* = 1 to 4) denotes the energy transfer upconversion coefficients from Yb^3+^ to the ^3^H_6_, ^3^F_4_, ^3^H_4_ and ^1^D_2_ states of Tm^3+^, respectively. *b*_*ij*_ is the branching ratios for the radiative transitions from the initial state *i* to the terminal state *j* of Tm^3+^ (*j* < *i*). ${\sigma }_{{{{{{\rm{p}}}}}}}^{a}$ is the absorption cross-section of Yb^3+^ at 975 nm. ${\sigma }_{{{{{{\rm{d}}}}}}}^{a}$ and ${\sigma }_{{{{{{\rm{d}}}}}}}^{{se}}$ represent the excited state absorption cross-section and stimulated emission cross-section, respectively, for the energy transfer process between ^1^G_4_ and ^1^I_6_. *v*_p_ and *v*_d_ denote the frequencies of 975-nm and 730-nm beams, respectively. *I*_p_ and *I*_d_ denote the intensities of 975-nm and 730-nm beams, respectively. *h* is Planck’s constant. *k*_et_ is the coefficient of energy transfer from the ^1^I_6_ state of Tm^3+^ to the ^6^P_7/2_ state of Gd^3+^. *φ*_gd_ represents the efficiency of the energy migration process through the Gd^3+^ sublattice. *n*_defect_ represents the effective population of surface quenchers. Here, similar to the energy transfer upconversion process, the SMED process can be regarded as the energy transfer from ^1^I_6_ state to surface quenchers through the Gd^3+^ sublattice, and written as the term ${k}_{{{{{{\rm{et}}}}}}}{\varphi }_{{{{{{\rm{gd}}}}}}}{n}_{{{{{{\rm{defect}}}}}}}{n}_{7}$. We defined the product of *k*_et_, *φ*_gd_ and *n*_defect_ as *M*_SEMD_ (s^−1^), which represents the equivalent energy dissipation rate of the SMED mechanism. Here, by referring to the experimental results, we estimated the value of *M*_SEMD_ for the cases of bare-core, inert shell and NaYF_4_-based Tm^3+^-activated systems. To achieve a high depletion efficiency of 95% for the bare-core nanoparticles, the value of *M*_SEMD_ is estimated to be at least 2.0 **×** 10^6 ^s^−1^. As for the inert-shell protected nanoparticles, the value of *M*_SEMD_ turns out to be 5.0 **×** 10^3 ^s^−1^, which is only 1/400 of the counterpart in bare-core nanoparticles. For the NaYF_4_-based Tm^3+^-doped systems, the value of *M*_SEMD_ is set to 0 s^−1^. The values of the parameters used in the numerical simulation were listed in Supplementary Table 2.

Based on the above rate equations, an increased decline in the population of the ^1^G_4_ state with increasing depletion intensity was obtained in our simulation, which accorded with our experimental results. Moreover, nanocomposites and nanostructures of particles would affect the depletion efficiency (e.g., inert shell, particle size and concentration of Gd^3+^ ions) since they can affect the value of *M*_SEMD_. Increasing *M*_SEMD_ enhanced the optical depletion efficiency of emission from the ^1^G_4_ state, and the representative results were shown in Fig. 3f and Supplementary Fig. 6.

### Theoretical analysis of the SMED mechanism

To better understand the proposed SMED depletion mechanism, a simplified four-level system (Supplementary Fig. 7) is used to investigate the relationship between optical depletion efficiency and the SMED process via steady-state rate equations:

11 $$\frac{d{n}_{s0}}{{dt}}=-\frac{d{n}_{{{{{{\rm{s}}}}}}1}}{{dt}}$$

12 $$\frac{d{n}_{{{{{{\rm{s}}}}}}1}}{{dt}}={\alpha }_{{{{{{\rm{p}}}}}}}{n}_{{{{{{\rm{s}}}}}}0}-({w}_{1}{n}_{0}+{w}_{2}{n}_{1}+{w}_{3}{n}_{2}){n}_{{{{{{\rm{s}}}}}}1}-\frac{{n}_{{{{{{\rm{s}}}}}}1}}{{\tau }_{{{{{{\rm{s}}}}}}1}}$$

13 $$\frac{d{n}_{0}}{{dt}}={\beta }_{1}{n}_{1}+\frac{{n}_{1}}{{\tau }_{1}}+\frac{{n}_{2}}{{\tau }_{2}}+\frac{{n}_{3}}{{\tau }_{3}}+{k}_{{{{{{\rm{et}}}}}}}{\varphi }_{{{{{{\rm{net}}}}}}}{n}_{{{{{{\rm{defect}}}}}}}{n}_{3}-{w}_{1}{n}_{{{{{{\rm{s}}}}}}1}{n}_{0}$$

14 $$\frac{d{n}_{1}}{{dt}}={\beta }_{2}{n}_{2}+{w}_{1}{n}_{{{{{{\rm{s}}}}}}1}{n}_{0}-{\beta }_{1}{n}_{1}-{w}_{2}{n}_{{{{{{\rm{s}}}}}}1}{n}_{1}-\frac{{n}_{1}}{{\tau }_{1}}-{\alpha }_{{{{{{\rm{d}}}}}}}{n}_{1}$$

15 $$\frac{d{n}_{2}}{{dt}}={\beta }_{3}{n}_{3}+{w}_{2}{n}_{{{{{{\rm{s}}}}}}1}{n}_{1}-{\beta }_{2}{n}_{2}-{w}_{3}{n}_{{{{{{\rm{s}}}}}}1}{n}_{2}-\frac{{n}_{2}}{{\tau }_{2}}$$

16 $$\frac{d{n}_{3}}{{dt}}={w}_{3}{n}_{{{{{{\rm{s}}}}}}1}{n}_{2}+{\alpha }_{{{{{{\rm{d}}}}}}}{n}_{1}-\frac{{n}_{3}}{{\tau }_{3}}-{\beta }_{3}{n}_{3}-{k}_{{{{{{\rm{et}}}}}}}{\varphi }_{{{{{{\rm{net}}}}}}}{n}_{{{{{{\rm{defect}}}}}}}{n}_{3}$$

In these equations, ${n}_{i}$ (*i* = 0 to 3) represents the population of activator ions at different energy states. Particularly, *n*_1_ is the population of ions at the emitting state of interest. *n*_s0_ and *n*_s1_ represent the population of sensitizer ions on the ground and excited states. *β*_*i*_ (*i* = 1 to 3) and *τ*_*i*_ (*i* = 1 to 3) represent the non-radiative decay rates and the radiative lifetimes of different energy states in activator ions. *w*_*i*_ (*i* = 1 to 3) denotes the energy transfer upconversion coefficients from sensitizers to activators, respectively. *α*_p_ and *α*_d_ represent the absorption rates for the excitation beam and depletion beam, respectively. *k*_et_ is the coefficient of energy transfer from the activators to the migrators. *φ*_net_ represents the energy migration efficiency of the sublattice network. *n*_defect_ represents the effective population of surface quenchers. Note that for a better understanding of the proposed mechanism, the excitation process has been simplified with the condition that the sensitizers existing in the luminescence system would not affect the calculation results.

Equations (14)–(16) can be rewritten as

17 $${n}_{1}=\frac{{\beta }_{2}{n}_{2}+{w}_{1}{n}_{{{{{{\rm{s}}}}}}1}{n}_{0}}{{w}_{2}{n}_{{{{{{\rm{s}}}}}}1}+\frac{1}{{\tau }_{1}}+{\beta }_{1}+{\alpha }_{{{{{{\rm{d}}}}}}}}$$

18 $${n}_{2}=\frac{{\beta }_{3}{n}_{3}+{w}_{2}{n}_{{{{{{\rm{s}}}}}}1}{n}_{1}}{{w}_{3}{n}_{{{{{{\rm{s}}}}}}1}+\frac{1}{{\tau }_{2}}+{\beta }_{2}}$$

19 $${n}_{3}=\frac{{\alpha }_{{{{{{\rm{d}}}}}}}{n}_{1}+{w}_{3}{n}_{{{{{{\rm{s}}}}}}1}{n}_{2}}{\frac{1}{{\tau }_{3}}+{\beta }_{3}+{k}_{{{{{{\rm{et}}}}}}}{\varphi }_{{{{{{\rm{net}}}}}}}{n}_{{{{{{\rm{defect}}}}}}}}$$

In a feasible SMED system, the upconversion processes from the emitting state to further higher-lying states should be weak. For example, in Tm^3+^-doped NaGdF_4_ system, the cross-relaxation processes ^1^G_4_ + ^3^H_4_ → ^1^D_2_ + ^3^F_4_ and ^3^H_4_ + ^1^G_4_ → ^1^D_2_ + ^3^F_4_ are too weak to transfer electrons from ^1^G_4_ state to ^1^D_2_ state; in Tb^3+^-doped NaGdF_4_ system, the excitation beam cannot even pump the electrons from the emitting state ^5^D_4_ to any higher-lying states. Therefore, the upconversion terms *w*_2_ *n*_s1_ *n*_1_ and *w*_3_ *n*_s1_ *n*_1_ can be neglected here.

Combining Eqs. (17) and (18), we have:

20 $${n}_{1}=\frac{\frac{{{\beta }_{2}\beta }_{3}{n}_{3}}{\frac{1}{{\tau }_{2}}+{\beta }_{2}}+{w}_{1}{n}_{{{{{{\rm{s}}}}}}1}{n}_{0}}{\frac{1}{{\tau }_{1}}+{\beta }_{1}+{\alpha }_{{{{{{\rm{d}}}}}}}}$$

With the combination of Eqs. (19) and (20), we could also get:

21 $${n}_{1}=\frac{\frac{{{\beta }_{2}\beta }_{3}{\alpha }_{{{{{{\rm{d}}}}}}}{n}_{1}}{\left(\frac{1}{{\tau }_{3}}+{\beta }_{3}+{k}_{{{{{{\rm{et}}}}}}}{\varphi }_{{{{{{\rm{net}}}}}}}{n}_{{{{{{\rm{defect}}}}}}}\right)\left(\frac{1}{{\tau }_{2}}+{\beta }_{2}\right)}+{w}_{1}{n}_{{{{{{\rm{s}}}}}}1}{n}_{0}}{\frac{1}{{\tau }_{1}}+{\beta }_{1}+{\alpha }_{{{{{{\rm{d}}}}}}}}$$

By rearranging Eq. (21), we have:

22 $${n}_{1}=\frac{{w}_{1}{n}_{{{{{{\rm{s}}}}}}1}{n}_{0}}{\frac{1}{{\tau }_{1}}+{\beta }_{1}+{\alpha }_{{{{{{\rm{d}}}}}}}\left(1-\frac{{{\beta }_{2}\beta }_{3}}{\left(\frac{1}{{\tau }_{3}}+{\beta }_{3}+{k}_{{{{{{\rm{et}}}}}}}{\varphi }_{{{{{{\rm{net}}}}}}}{n}_{{{{{{\rm{defect}}}}}}}\right)\left(\frac{1}{{\tau }_{2}}+{\beta }_{2}\right)}\right)}$$

Equation (22) has expressed the correlation of the population *n*_1_ and the absorption rate of the depletion beam *α*_d_, which follows a simple functional relationship $y=a/(b+{cx})$. Here, *y* denotes the population of the emitting state. *x* denotes the intensity of the depletion laser beam. The variable *a* mainly represents the factor of energy excitation processes involved in the emitting state. The variable *b* represents the factor of intrinsic energy loss pathways involved in the emitting state. The variable *c* mainly represents the factor of energy states which participate in the depletion processes, and typically the state accepts the losing energy from the emitting state. According to this equation, if the intensity of the depletion beam increases, the population of the emitting state will be effectively reduced. Moreover, increasing the value of *c* would relieve the required depletion intensity for a specific depletion efficiency. Here, the parameters *k*_et_, *φ*_net_ and *n*_defect_ can substantially contribute to the value of *c*. In other words, the higher the energy transfer rate is from activators to migrators, the more efficient the energy migration process occurs. As a result, more surface quenchers would help lower the saturation intensity significantly, which is in accord with the experimental observation. Hence, the SMED mechanism is a powerful strategy for achieving a highly efficient emission depletion process.

### Density functional theory (DFT) calculations

We performed first-principles calculations based on density functional theory (DFT) using the Vienna ab initio simulation package with the projector augmented wave method^36,56^. The exchange-correlation interactions were approximated by the Perdew–Burke–Ernzerh generalized gradient approximation (GGA-PBE)^57^. To precisely map the position of the localized 4 f orbital of gadolinium ions, the screened-exchange hybrid density functional HSE06 with 5% Hartree–Fock (HF) exchange interaction was employed^58,59^. The kinetic energy cut-off of the plane wave was set to 520 eV, the energy convergence criterion was 1 × 10^−4^, and the maximum force on each relaxed atom was less than 0.02 eV/Å. For the surface model, a 15-Å vacuum spacer was introduced to separate the 9-layer YF-terminated (0001) surface. Note that we chose NaYF_4_ lattice as model systems instead of NaGdF_4_ because quantum calculation involving a large quantity of 4 f electrons could be highly resource-demanding. Given the same hexagonal space group, the electronic structure of Gd dopants in the NaYF_4_ lattice should resemble the host Gd ions in the NaGdF_4_ lattice.

We studied the influence of defects on the electronic structure of Gd-doped NaYF_4_ nanocrystal using both bulk and slab models that represent the interior area and the surface of a given nanoparticle, respectively. The defects of interest were fluorine vacancy (V_F_), oxygen substitution (O_F_ and O_2F_), hydroxyl substitution (OH_F_), and water adsorbed on Gd (H_2_O-Gd), as well as OH adsorbed on Gd (OH-Gd), which could be formed during nanoparticle synthesis. The calculated density of states (DOS) showed that the NaYF_4_ host has a clean bandgap of 7.74 eV and the Gd dopant barely affects the electronic structure of the system except for 4f-based midgap states. Here, we used the energy bandgap between occupied and empty 4 f orbitals to mimic the energy difference between the Gd’s ground state (^3^S_7/2_) and its first excited state (^6^P_7/2_). Note that the calculated 4f–4 f gap is overestimated compared to the ^3^S_7/2_−^6^P_7/2_ gap because DFT calculation can only access the ground-state electronic structure of the given system and the spin-orbit coupling was not considered. Despite the quantitative discrepancy between simulated and experimentally measured ^3^S_7/2_−^6^P_7/2_ gap, it is rational to use the theoretically captured electronic changes in NaYF_4_-based systems containing different defects for the mechanistic explanation.

As the total and projected density of states of NaYF_4_:Gd and NaYF_4_:Gd+O_2F_ shown in Supplementary Fig. 8a, b, considering that the values of these energy gaps are close to the 4f-4f gap of the Gd^3+^ dopant, Förster resonance energy transfer from excited Gd^3+^ ions to defect sites can occur via dipole-dipole coupling. The occupied state and empty states mainly originate from O’s p orbital and Y’s 4d orbital (Supplementary Fig. 8c, d), respectively, suggesting dipole-allowed electronic transitions between the states of high oscillation strength. Additionally, these impurity-state-involved transitions should have broadband absorption that ensures a large spectral overlap with the Gd^3+^ emission. Moreover, our calculations suggested that these lattice defects prefer proximity to Gd^3+^ dopant, indicating a short Gd-defect distance. This implies that energy transfer from Gd^3+^ ions to defect quenchers can occur at a high transfer rate, in agreement with the *M*_SMED_ calculation in the aforementioned rate equation modeling. On the other hand, as the total and projected density of states of the hydroxyl- and water-adsorbed NaYF_4_:Gd (0001) surface shown in Supplementary Fig. 8e, f, although adsorption of OH^−^ or H_2_O introduced midgap states, the energy differences between these states and host states do not match the ^3^S_7/2_−^6^P_7/2_ gap, suggesting that electronic deactivation of excited Gd^3+^ ions via absorbed moieties is trivial. These adsorbates likely deactivate excited states of Gd^3+^ or Tm^3+^ directly through inefficient multiphonon coupling. Supplementary Fig. 8g summarizes the optically active and inert defects for the depletion of Gd^3+^ ions.

### Software

Software Olympus FV10 ASW ver. 4.0a was used to control laser scanning microscopy imaging and acquire images. Software Ocean Optics SpectraSuite was used to control spectrometer and acquire spectra. No custom algorithms or software that are central to the data collection. Matlab (v2019a) was used for the analysis of spectra, the analysis of microscopic images and the calculation of numerical simulations. FRC analysis was performed with the code provided by previous work^60^. The first principles calculations based on DFT were performed using the Vienna ab initio simulation package with the projector augmented wave method^36,56^.

### Reporting summary

Further information on research design is available in the Nature Research Reporting Summary linked to this article.

## Supplementary information

Supplementary Information

Reporting Summary

## Data Availability

The data generated in this study supporting the plots in Figs. 1–5 are provided in the Source data files. Additional data are available from the corresponding authors upon request. Source data are provided with this paper.

## Source data

Source Data

## Code availability

The codes for theoretical modeling and numerical simulations are available from the corresponding authors on request.
