# Supplementary material for: Super-resolution microscopy enabled by high-efficiency surface-migration emission depletion
Source: Nat Commun. 2022 Nov 4;13:6636. doi: 10.1038/s41467-022-33726-7 (PMC9636245; doi:10.1038/s41467-022-33726-7)
Supplement: Supplementary file 1 — Supplementary Information [file 41467_2022_33726_MOESM1_ESM.pdf]

Supplementary information for

Super-resolution microscopy enabled by high-efficiency surface-migration emission depletion

Rui Pu[1†], Qiuqiang Zhan[1,2,3†*], Xingyun Peng[1], Siying Liu[1], Xin Guo[1], Liangliang Liang[4], Xian Qin[4], Ziqing Winston Zhao[4,5,6], and Xiaogang Liu[4,7*]

[1]Centre for Optical and Electromagnetic Research, South China Academy of Advanced Optoelectronics, South China Normal University, Guangzhou 510006, P. R. China.

[2]National Center for International Research on Green Optoelectronics, Guangdong Engineering Research Centre of Optoelectronic Intelligent Information Perception, South China Normal University, Guangzhou 510006, P. R. China.

[3]MOE Key laboratory & Guangdong Provincial Key laboratory of Laser Life Science, South China Normal University, Guangzhou 510631, P. R. China.

[4] Department of Chemistry, National University of Singapore, 3 Science Drive 3, Singapore 117543, Singapore.

[5]Centre for BioImaging Sciences, National University of Singapore, 14 Science Drive 4, Singapore 117557, Singapore

[6]Mechanobiology Institute, National University of Singapore, 5A Engineering Drive 1, Singapore 117411, Singapore

[7]The N.1 Institute for Health, National University of Singapore, Singapore 117456, Singapore.

[†]These authors contributed equally: Rui Pu, Qiuqiang Zhan

*Corresponding authors, ✉email: zhanqiuqiang@m.scnu.edu.cn (Q.Z.); chmlx@nus.edu.sg (X.L.).

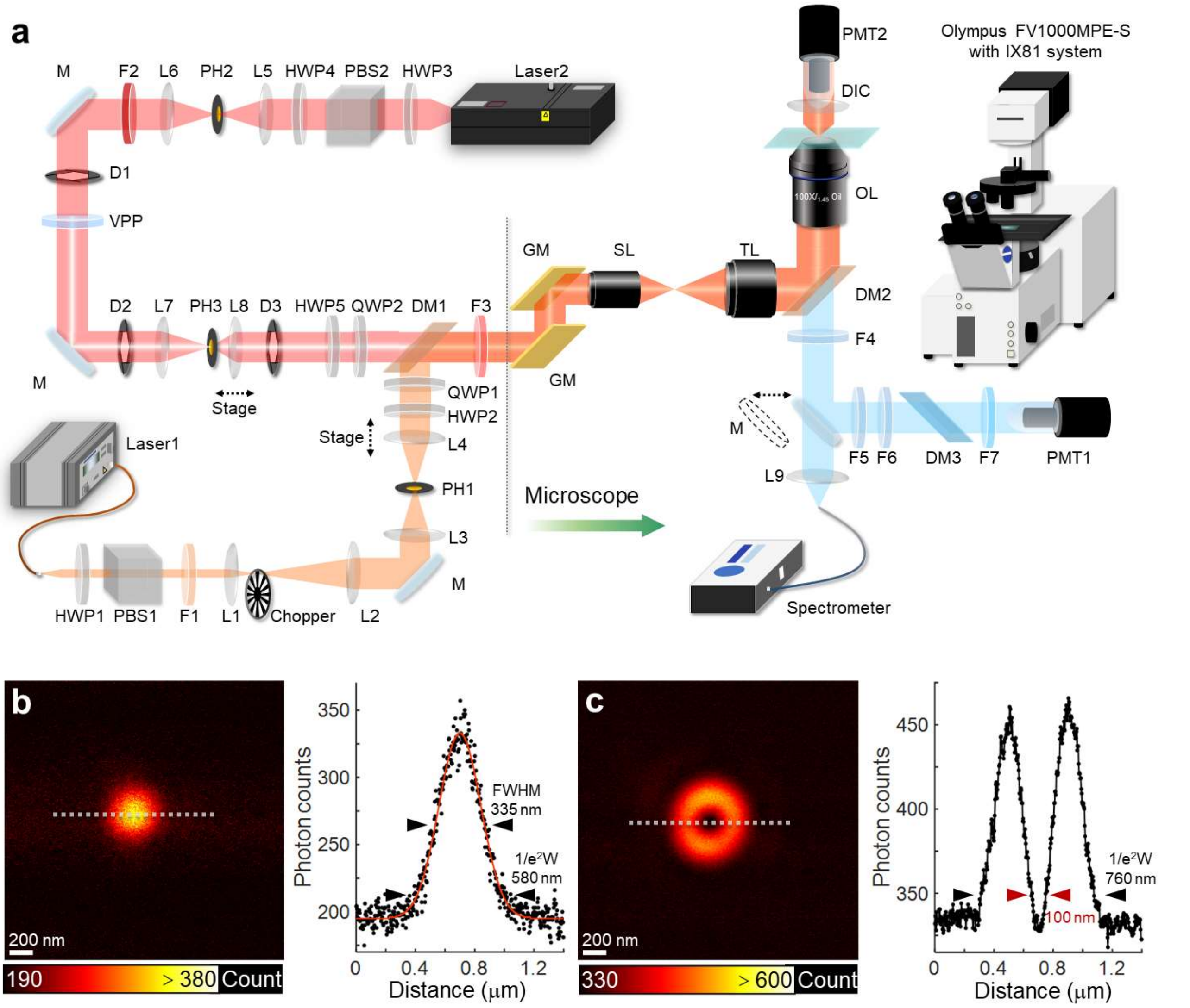

Supplementary Fig. 1 a, Schematic of the optical spectroscopy/microscopy system used for super-resolution imaging. M: silver reflection mirrors. F1: 975-nm laser band-pass filter; F2: 730-nm band-pass filter; F3: 715-nm long-pass filter; F4: 694-nm short-pass filter; F5: 720-nm short-pass filter; F6: 665-nm short-pass filter; F7: 480-nm band-pass filter; DM1: 950-nm short-pass dichroic mirror; DM2: 690-nm short-pass dichroic mirror; DM3: 458-nm long-pass dichroic mirror; L1, L8: 25-mm focus length lenses; L2: 100-mm focus length lens; L3, L4, L5, L6, L7: 50-mm focus length lenses; L4 and L8 were controlled with three-dimensional displacement stages; PH1, PH2: 25-μm gold-coated pinholes; PH3: 50-μm gold-coated pinhole; D1-3: Diaphragms. HWP1-HWP5: half-wave plates; QWP1-2: quarter-wave plates; PBS1-2: polarization beam splitters; VPP: 730-nm vortex phase plate; OL: 100×oil immersed objective lens (NA=1.45); PMT1, PMT2: photomultiplier tubes; GM: Galvanometer scan gold-coated mirrors; SL: scan lens; TL: tube lens; DIC: differential interference contrast. **b**, The imaging spot and corresponding line profile of the CW 975-nm excitation PSF, imaged using single gold nanoparticle (120 nm in diameter). I_{975} = 76 W cm^{-2}; Image dimensions: 800 × 800 pixels; pixel size: 3 nm; pixel dwell time: 100 μs. The image was generated by repeating the image acquisition five times and taking the average. **c**, The imaging spot and corresponding line profile of the CW 730 nm doughnut depletion PSF. P_{730} = 10 μW; Image dimensions: 800 × 800 pixels; pixel size: 3 nm; pixel dwell time: 2 μs. The image was generated by repeating the image acquisition twenty times and taking the average.

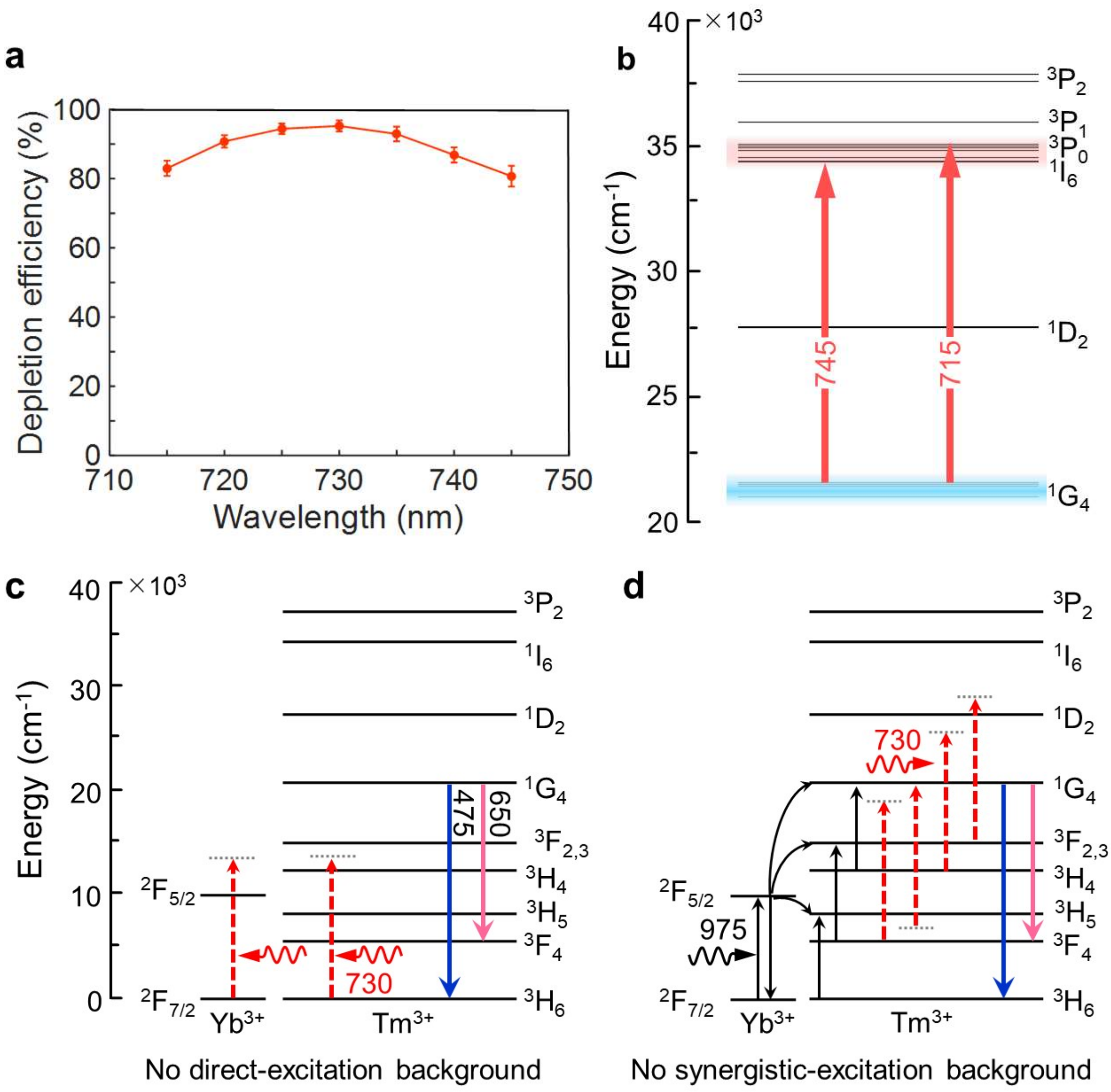

Supplementary Fig. 2 Characterization of depletion wavelength matching. **a**, Depleting-wavelength dependent depletion efficiency of $NaGdF_4$:Yb/Tm (18/0.3%) nanoparticles. The intensities of the excitation beam and depletion beam were kept at 21.7 kW cm^{-2} and 3.01 MW cm^{-2}, respectively. Data were presented as mean value ± standard deviation (SD). Error bars are defined as the SD of n = 3 independent measurements. **b**, The broad working range of the depletion beam showing the coincidence with the broadband ESA from the $^1G_4$ state to the $^1I_6$ state of Tm^{3+} ion[1,2]. **c**, **d**, Schematic energy diagrams illustrating the mechanism of no re-excitation background involving SMED microscopy. Since the 730 nm photon cannot initiate ground-state absorption for Yb^{3+} and Tm^{3+} ions (**c**), this depletion beam would not induce a direct-excitation emission background, which, however, is an inherent problem in traditional STED microscopy[3,4]. In company with the 975 nm excitation beam, the 730 nm beam excitation also does not match any excited-state absorption transition of Tm^{3+} to populate its emitting $^1G_4$ state (**d**). Therefore, the addition of the 730 nm depletion beam will not generate synergistic-excitation background emission[4,5].

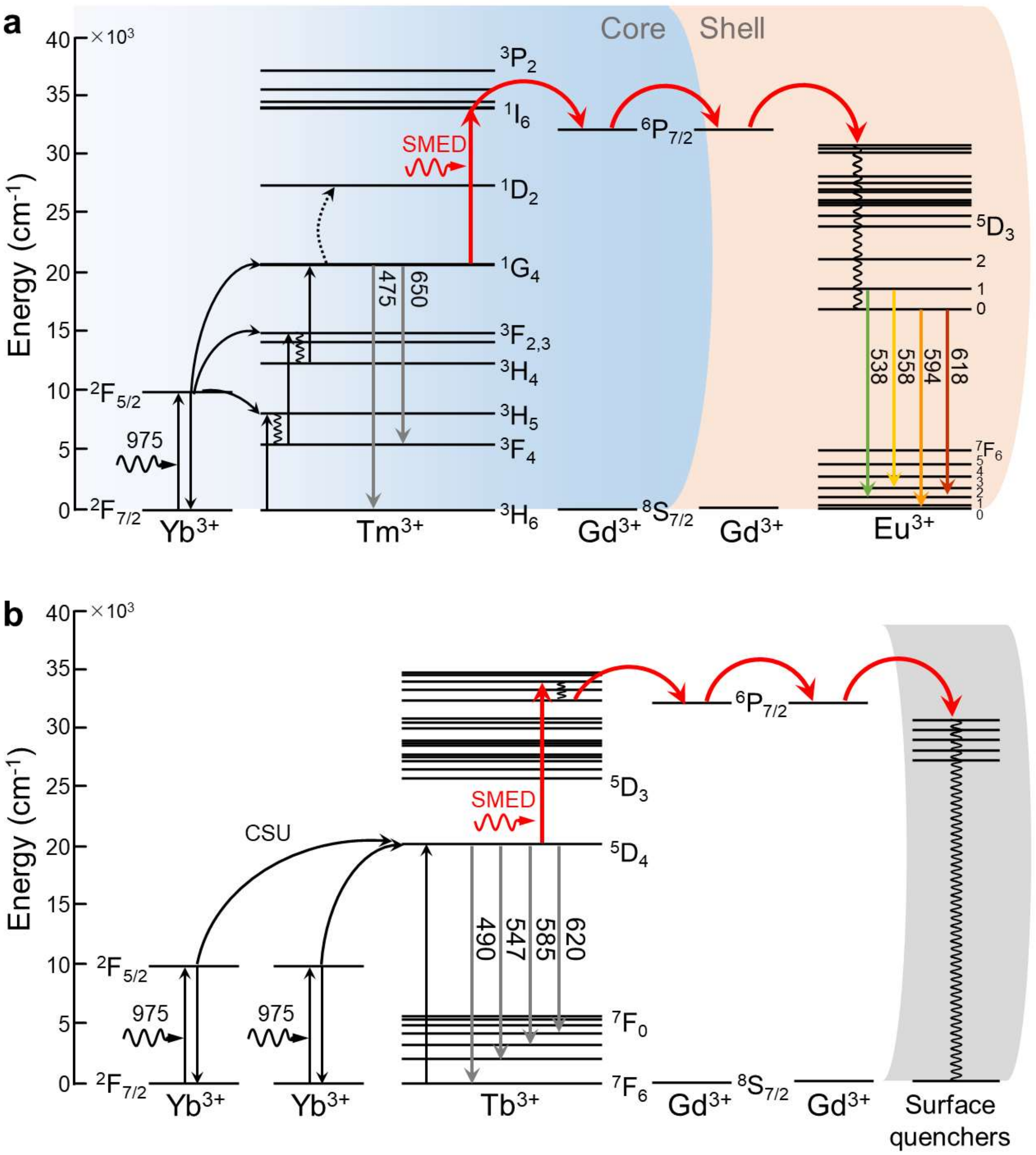

Supplementary Fig. 3 a, Schematic energy diagrams for the proposed energy transfer pathways within $NaGdF_4$:Yb/Tm (18/0.3%)@$NaGdF_4$: Eu (15%) core-shell nanoparticles. When co-excited with a 730-nm depletion beam, the excitation energy at the $^1G_4$ state was transferred to higher-lying states and then captured by the Gd^{3+} sublattice. The Eu^{3+} ions doped in the shell layer absorb the overflowing energy from Gd^{3+}, thereby enhancing Eu^{3+} emission[6,7]. **b**, Proposed SMED mechanism for Tb^{3+}-activated $NaGdF_4$:Yb/Tb (12/8%) nanoparticles.

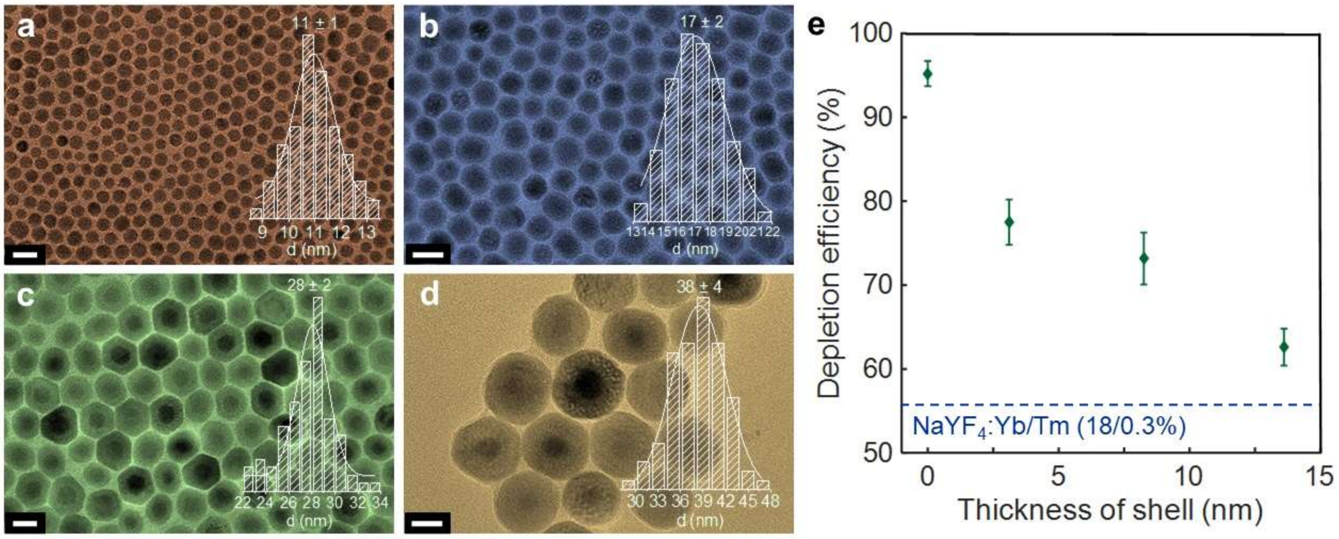

Supplementary Fig. 4 Comparison of depletion efficiencies of $NaGdF_4$:Yb/Tm (18/0.3%)@$NaYF_4$ core-shell nanoparticles with different shell thicknesses. a, TEM image of $NaGdF_4$:Yb/Tm (18/0.3%) nanoparticles, average size: 11 ± 1 nm in diameter. Scale bar: 20 nm. **b**, TEM image of $NaGdF_4$:Yb/Tm (18/0.3%)@$NaYF_4$ nanoparticles, average size: 17 ± 2 nm in diameter. Shell thickness: 3 nm. Scale bar: 20 nm. **c**, TEM image of $NaGdF_4$:Yb/Tm (18/0.3%)@$NaYF_4$ nanoparticles, average size: 28 ± 2 nm in diameter. Shell thickness: 8.5 nm. Scale bar: 20 nm. **d**, TEM image of $NaGdF_4$:Yb/Tm (18/0.3%)@$NaYF_4$ nanoparticles, average size: 38 ± 4 nm in diameter. Shell thickness: 13.5 nm. Scale bar: 20 nm. (These inner-shell protected nanoparticles were all synthesized based on the 11 nm core one. The thickness of inert-shell is calculated by $t = (d - d_0)/2$, where d_0 denotes the diameter of core nanoparticles). e, Plot of depletion efficiency versus inert-shell thickness. The depletion efficiency decreases with increasing inert-shell thickness and gradually approaches the value of the $NaYF_4$:Yb/Tm (18/0.3%) sample. (co-irradiated by 975-nm CW beam (21.7 kW cm^{-2}) and 730-nm CW beam (3.01 MW cm^{-2}). The dotted line represents the depletion efficiency of $NaYF_4$:Yb/Tm (18/0.3%) nanoparticles.) According to the definition in numerical simulation of SMED mechanism (See methods for details), a thick inert-shell can suppress the surface effect[8], leading to a reduced value of n_{defect} and a decreased value of M_{SMED}. This observed decline in depletion efficiency accords well with the simulation. Data were presented as mean value ± SD. Error bars are defined as the SD of n = 3 independent measurements.

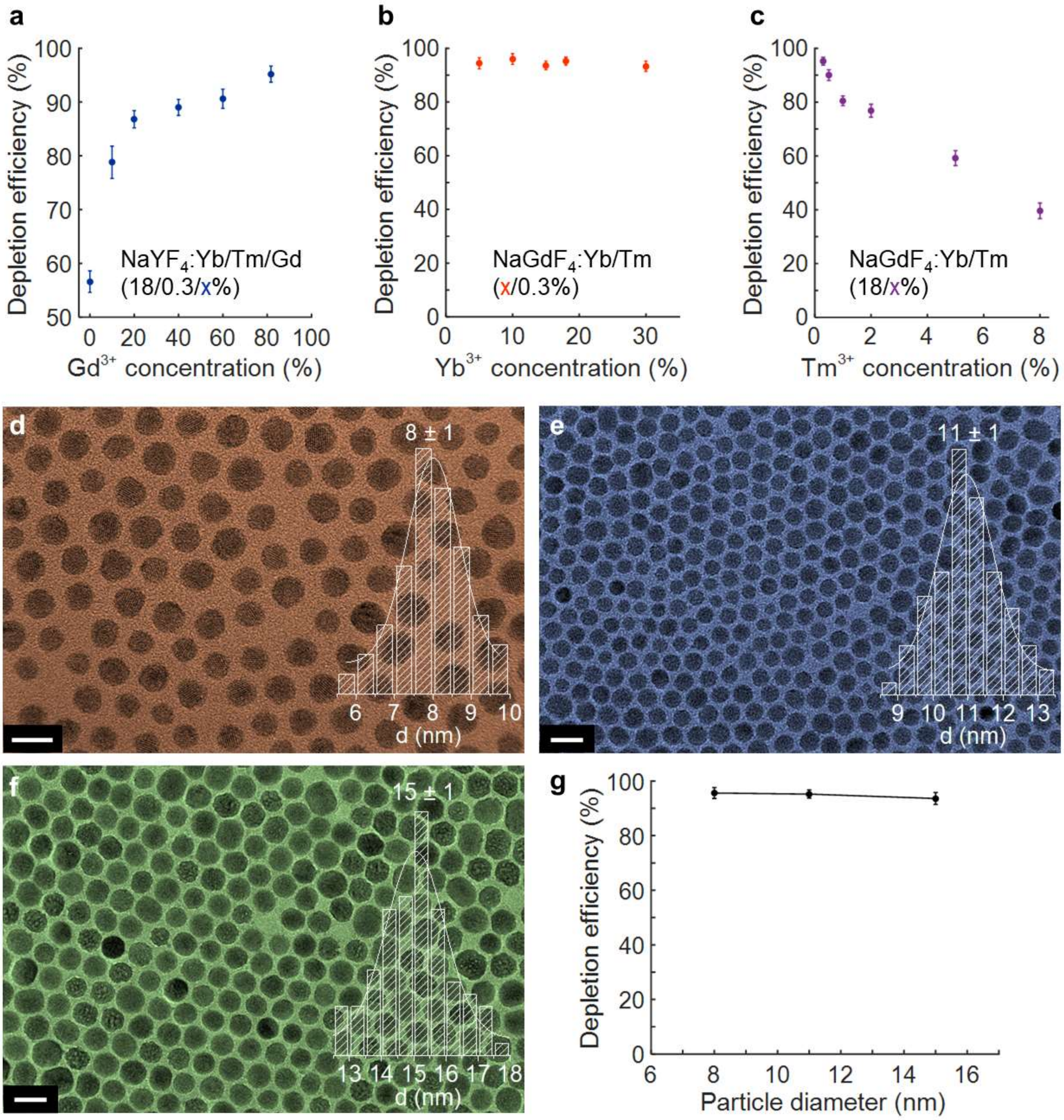

Supplementary Fig. 5 Depletion efficiencies measured for NaGdF$_4$:Yb/Tm nanoparticles with different doping concentrations and sizes. a, Depletion efficiencies of NaYF$_4$:Yb/Tm/Gd (18/0.3/x%) nanoparticles with x = 0, 10, 20, 40, 60 and 81.7 (NaGdF$_4$ host). Upon increasing Gd^{3+} concentration, the depletion efficiency was enhanced, suggesting that the enhanced Gd^{3+} ions interaction facilitates energy migration to surface quenchers[7]. Thus, a large φ_{gd} is essential to achieve a large M_{SMED} in the optical depletion process. **b**, Depletion efficiencies of NaGdF$_4$:Yb/Tm (x/0.3%) nanoparticles with x = 5, 10, 15, 18, and 30. The concentration of Yb^{3+} ions does not impact the depletion efficiencies of nanoparticles with high Gd^{3+} content. **c**, Depletion efficiencies of NaGdF$_4$:Yb/Tm (18/x%) nanoparticles with x = 0.3, 0.5, 2, 5 and 8. Interestingly, the depletion efficiency was also found to be dependent on the concentration of Tm^{3+}. The emission depletion phenomenon was observed for all samples, and the depletion efficiencies fall off upon increasing the Tm^{3+} concentration. This result is probably due to the cross-relaxation (CR) processes: $^1G_4 + {}^3H_4 \rightarrow {}^1D_2 + {}^3F_4$ and $^3H_4 + {}^1G_4 \rightarrow {}^1D_2 + {}^3F_4$, since these concentration-dependent CR processes can transfer many electrons from the emitting state $^1G_4$ to higher-lying states[9,10]. This typically

leads to a gradually boosted stimulated emission process from a higher-lying state to $^1G_4$ triggered by a 730-nm depletion beam. This stimulated emission process can partially counteract the ESA process and consequently compromise the depletion performance. **d-f**, TEM images of the $NaGdF_4$:Yb/Tm (18/0.3%) nanoparticles with average size: 8 ± 1 nm in diameter. Scale bar: 10 nm. (**d**), average size: 11 ± 1 nm in diameter. Scale bar: 20 nm. (**e**) and average size: 15 ± 1 nm in diameter. Scale bar: 20 nm. (**f**). **g**, Plot of depletion efficiency versus particle size. The reduction in depletion efficiency with increasing particle size is negligible. All the samples were tested under CW excitation at 975 nm (21.7 kW cm^{-2}) and CW depletion at 730 nm (3.01 MW cm^{-2}). Data in **a**, **b**, **c**, **g** were presented as mean value ± SD. Error bars are defined as the SD of n = 3 independent measurements.

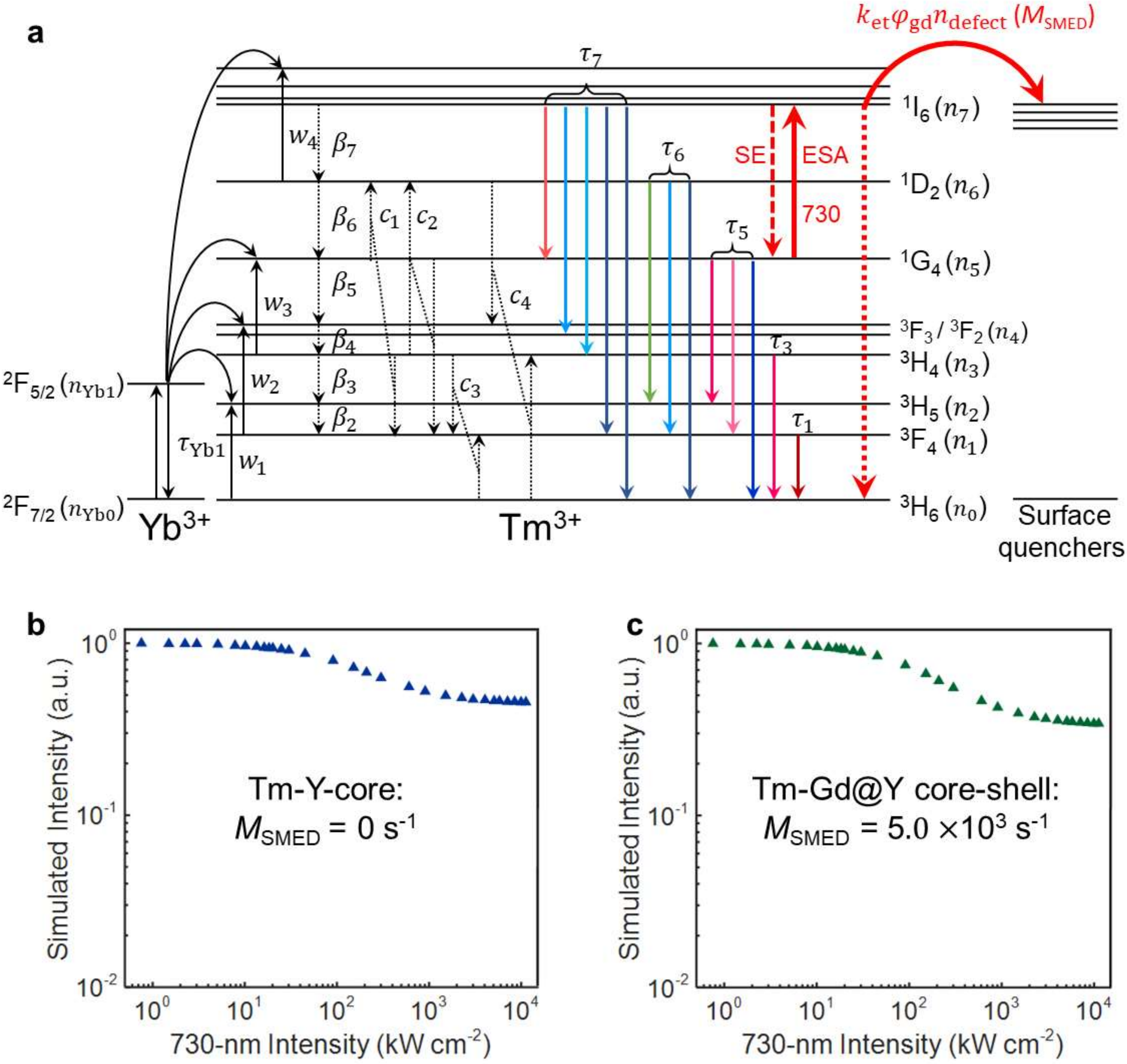

Supplementary Fig. 6 Simulation of the emission depletion in NaGdF$_4$:Yb/Tm with SMED mechanism. **a**, Schematic energy diagram for illustrating the rate equations in numerical simulation of the optical depletion mechanism in NaGdF$_4$:Yb/Tm system. **b**, **c**, Simulation of intensity-dependent depletion efficiencies of NaYF$_4$:Yb/Tm ($\varphi_{gd} = 0$, the value of M_{SMED} is 0) and NaGdF$_4$:Yb/Tm@NaYF$_4$, respectively. The suppressed surface effect leads to ultra-low levels of accessible n_{defect} and the value of M_{SMED} is 5.0×10^3 s^{-1}.

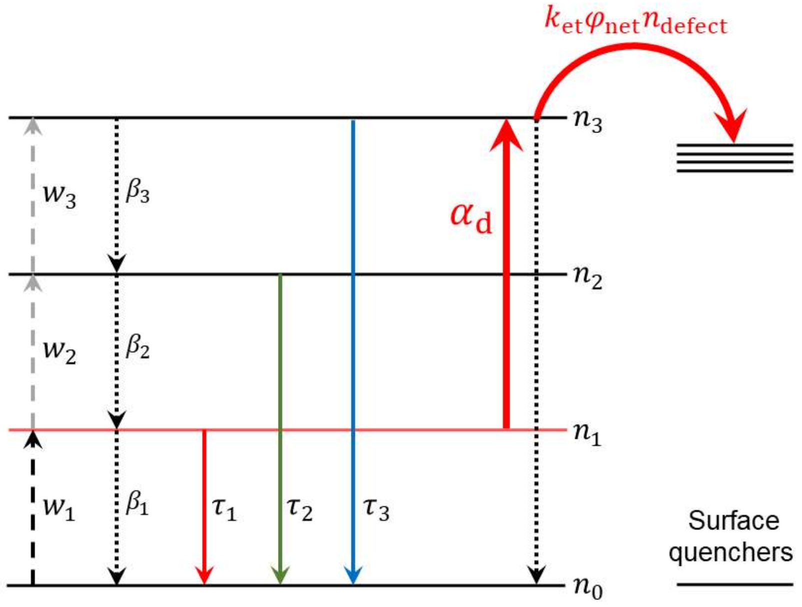

Supplementary Fig. 7 Energy diagram depicting the model of the proposed SMED mechanism.

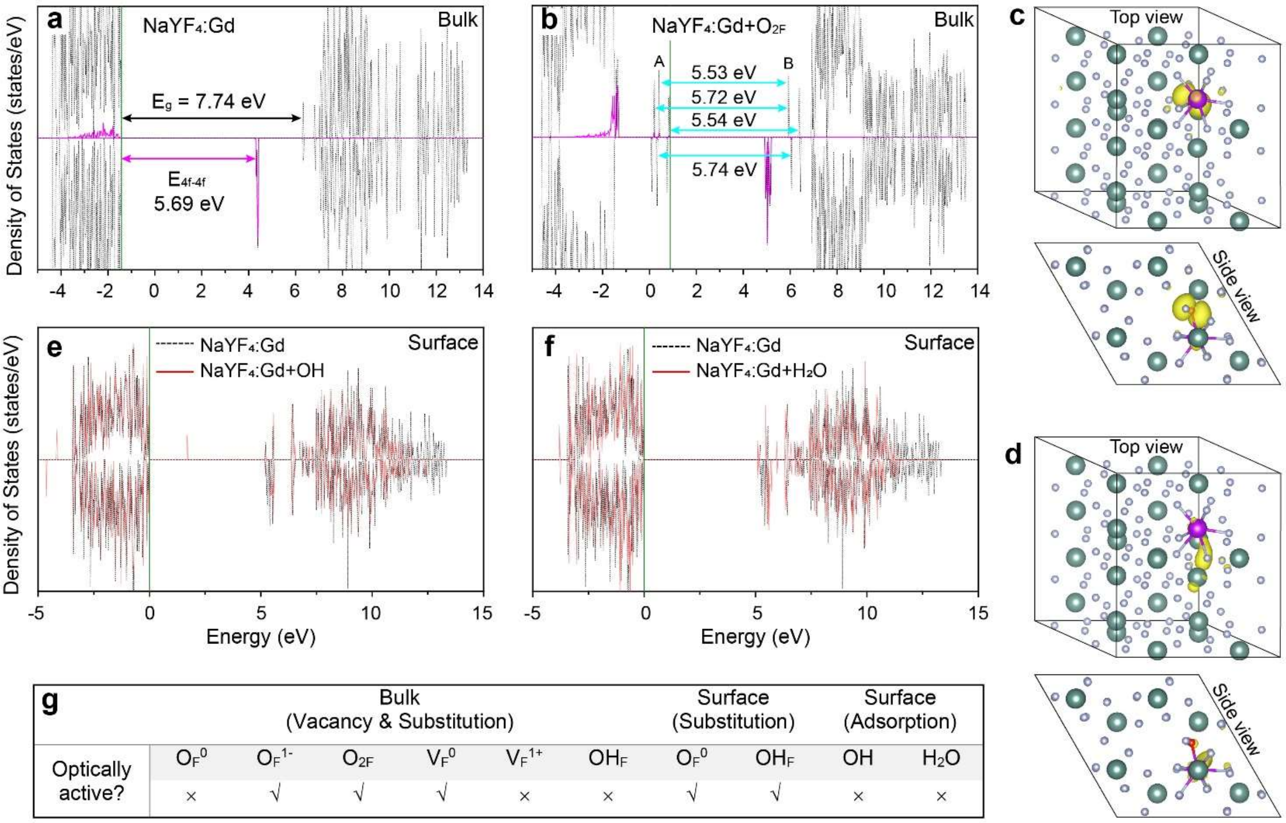

g	Bulk (Vacancy & Substitution)						Surface (Substitution)		Surface (Adsorption)	
Optically active?	O_F^0	O_F^{1-}	O_{2F}	V_F^0	V_F^{1+}	OH_F	O_F^0	OH_F	OH	H_2O
	×	√	√	√	×	×	√	√	×	×

Supplementary Fig. 8 Ground-state electronic structures of Gd^{3+}-doped $NaYF_4$ comprising different types of defects. a, b, Total and projected density of states of $NaYF_4$:Gd and $NaYF_4$:Gd+O_{2F}, respectively. O_{2F} defect denotes the substitution of two fluorine atoms with one oxygen atom. The blue arrows show the energy gaps between defect-associated midgap levels. **c**,**d**, Plotted charge distributions of two impurity states (labeled as A and B in **b**). **e**,**f**, Total and projected density of states of the hydroxyl- and water-adsorbed $NaYF_4$:Gd (0001) surface, respectively. **g**, Compiled different types of defects. √ and × indicate optically active and inert defects in the SMED mechanism, respectively.

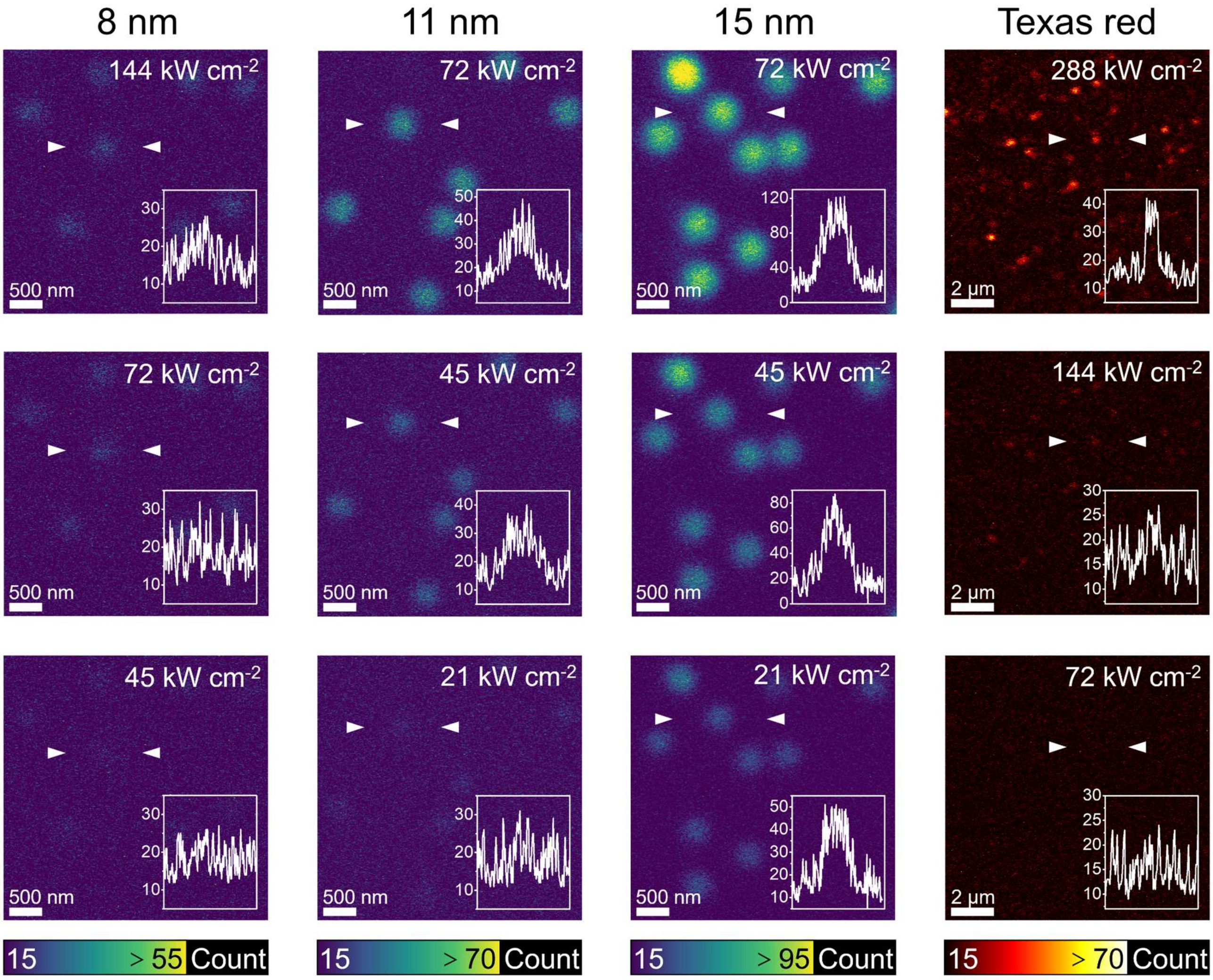

Supplementary Fig. 9 The brightness comparison of lanthanide-doped nanoparticles and organic fluorophore. Single-nanoparticles imaging of $NaGdF_4$: Yb/Tm (18/0.3%) nanoparticles with different sizes was performed under CW 975-nm excitation at different intensities. Image dimensions: 512 × 512 pixels; pixel size: 8 nm; pixel dwell time: 200 µs; acquisition time: 55 s. The two-photon excitation microscopic imaging for Texas Red molecules or small agglomerates was collected under the excitation of 830-nm femtosecond pulses[11]. Image dimensions: 512 × 512 pixels; pixel size: 24 nm; pixel dwell time: 200 µs; acquisition time: 55 s. More than three independent samples of nanoparticles of different size were used for this experiment with similar results obtained.

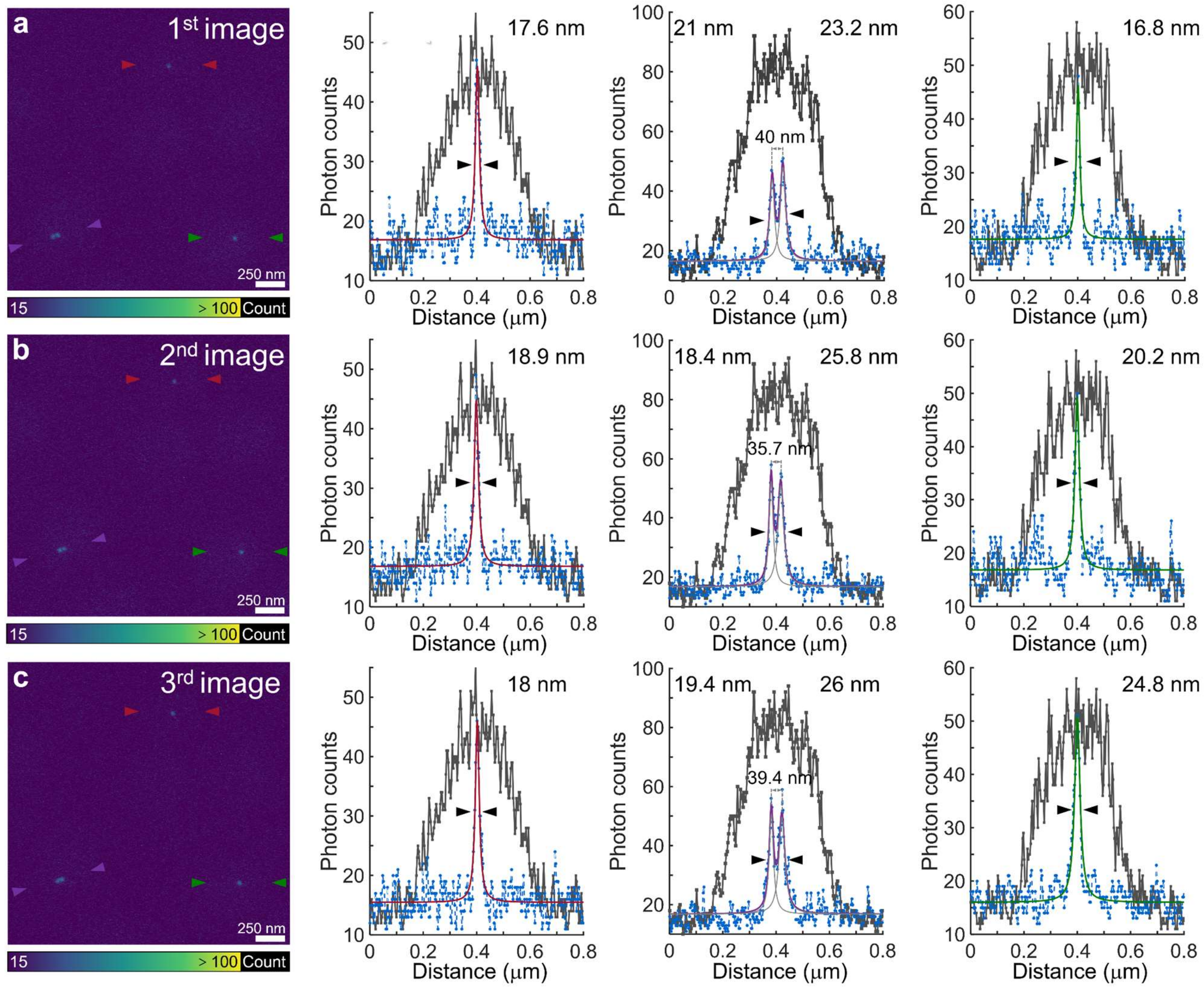

Supplementary Fig. 10 a, Single-nanoparticle super-resolution image and corresponding intensity profiles analysis which have been presented in Fig. 4. **b**,**c**, Two additional super-resolution images of the same area under the same testing condition and corresponding intensity profiles analysis. Images of **a-c** captured from three independent imaging experiments with similar results obtained.

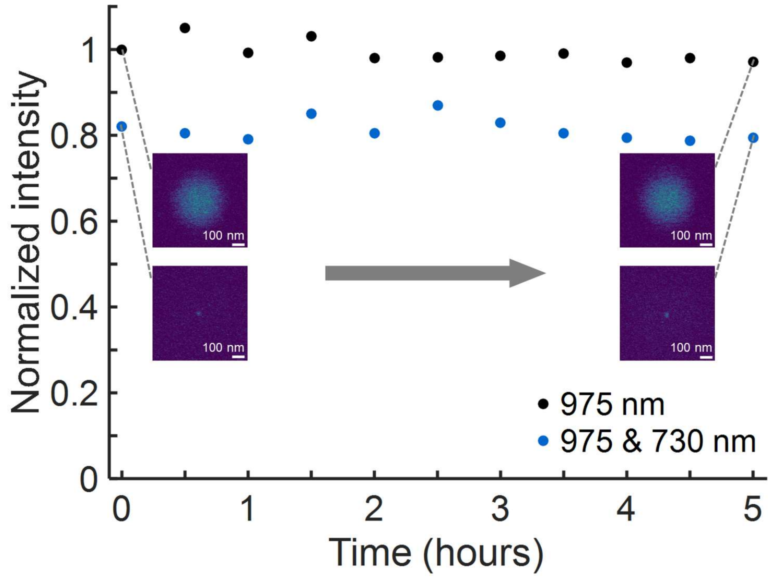

Supplementary Fig. 11 Photostability of SMED probe during laser scanning imaging. The change of emission intensity of $NaGdF_4$:Yb/Tm (18/0.3%) nanoparticles under irradiation with a 975-nm Gaussian beam or under co-irradiation with 975-nm Gaussian beam/730-nm doughut-shaped beam over a period of 5 h. I_{975} = 98 kW cm^{-2}, I_{730} = 1.09 MW cm^{-2}. The peak intensity of nanoparticle was recorded per 30 min. The position of the sample and the focal plane of objective lens were calibrated once data collection.

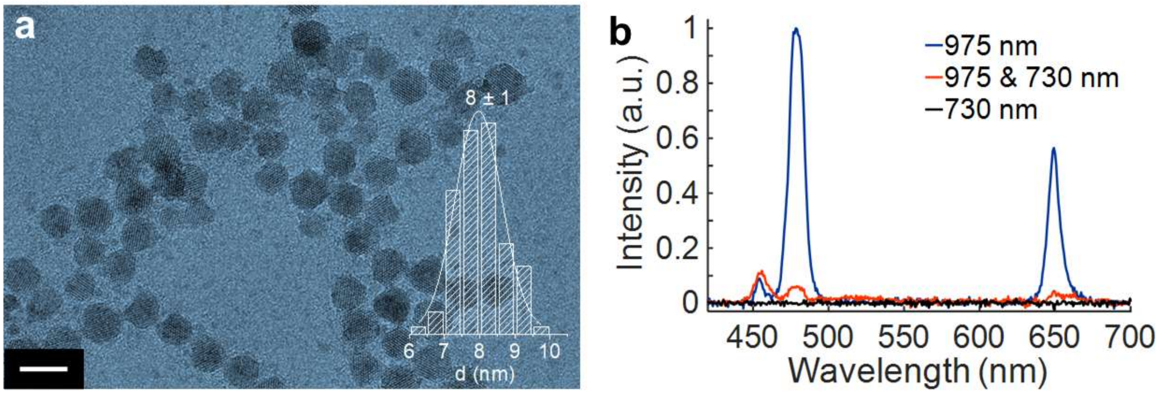

Supplementary Fig. 12 Characterization of bioconjugated lanthanide-doped nanoprobes. a, **b**, The representative TEM image and the depletion performance of Phalloidin-functionalized $NaGdF_4$:Yb/Tm (18/0.3%) nanoprobes (8±1 nm), scale bar: 10 nm. I_{975} = 21.7 kW cm^{-2}; I_{730} = 3.01 MW cm^{-2}.

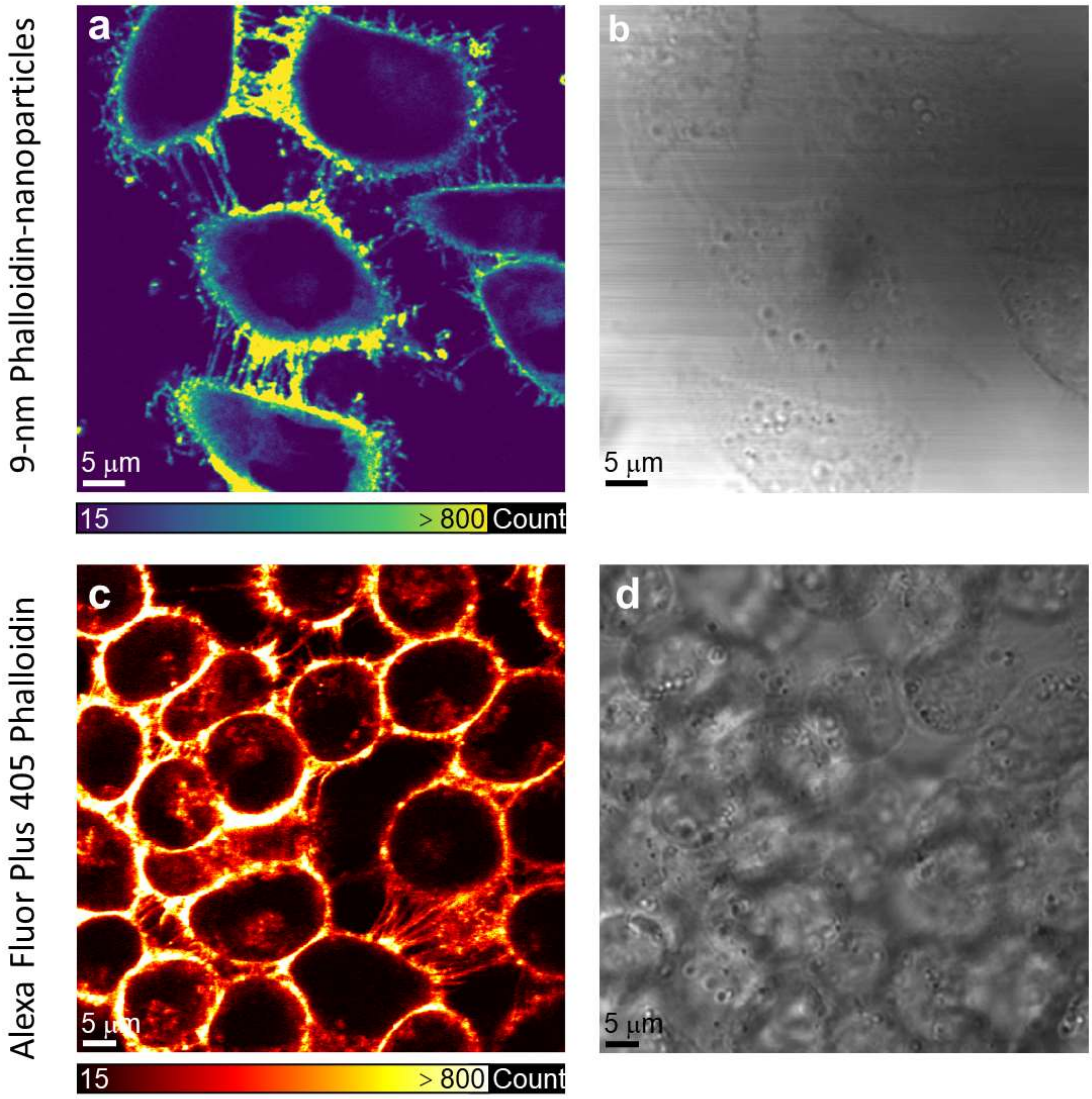

Supplementary Fig. 13 fluorescence and bright-field imaging of cancer cell. a, standard upconversion laser-scanning microscopic imaging under 975 nm-excitation of fixed HeLa cells stained with Phalloidin-conjugated 8-nm $NaGdF_4$:Yb/Tm (18/0.3%) nanoprobes. **b**, the corresponding bright field imaging of the sample shown in **a**. Image dimensions: 1024 × 1024 pixels; pixel size: 62 nm; pixel dwell time: 200 µs; acquisition time: 213 s. **c**, Two-photon laser-scanning microscopic imaging under 850 nm femtosecond laser excitation (310 kW cm^{-2}) of fixed HeLa cells stained with Alexa Fluor Plus 405 Phalloidin. **d**, Corresponding bright field imaging of the sample shown in **c**. Image dimensions: 512 × 512 pixels; pixel size: 138 nm pixel dwell time: 200 µs; acquisition time: 55 s. More than three independent labelling and imaging experiments show the similar results.

Supplementary Table 1 List of the as-synthesized samples employed in this study

Samples	No. of Figure	Measurements
$NaGdF_4$:Yb/Tm (18/0.3%) (11 ± 1 nm)	Fig. 1, 2, 3d, 3e; Extended Data Fig. 2	Emission depletion of various Tm-doped nanoparticles
$NaGdF_4$:Yb/Tm (18/0.3%)@ $NaYF_4$ (38 ± 4 nm)	Fig. 3b, d, e	
$NaYF_4$:Yb/Tm (18/0.3%)	Fig. 3c-e	
$NaGdF_4$:Yb/Tm(18/0.3%)@$NaGdF_4$: Eu (15%)	Fig. 3a	
$NaGdF_4$:Yb/Tm (18/0.3%) (11 ± 1 nm)	Fig. 4a-h Extended Data Fig. 10, 11	Super-resolution imaging
$NaGdF_4$:Yb/Tm (18/0.3%) (8 ± 1 nm)	Fig. 5a-i; Extended Data Fig. 12, 13a, b	
$NaGdF_4$:Yb/Tm (18/0.3%) (11 ± 1 nm)	Extended Data Fig. 5, 9	Dependence of brightness and emission depletion on the size and morphology
$NaGdF_4$:Yb/Tm (18/0.3%) (15 ± 1 nm)	Extended Data Fig. 5, 9	
$NaGdF_4$:Yb/Tm (18/0.3%) (8 ± 1 nm)	Extended Data Fig. 5, 9	
$NaGdF_4$:Yb/Tm (18/0.3%) (11 ± 1 nm)	Extended Data Fig. 4a, e	Dependence of depletion on the thickness of the inert shell
$NaGdF_4$:Yb/Tm (18/0.3%)@ $NaYF_4$ (17 ± 2 nm)	Extended Data Fig. 4b, e	
$NaGdF_4$:Yb/Tm (18/0.3%)@ $NaYF_4$ (28 ± 2 nm)	Extended Data Fig. 4c, e	
$NaGdF_4$:Yb/Tm (18/0.3%)@ $NaYF_4$ (38 ± 4 nm)	Extended Data Fig. 4d, e	
$NaYF_4$:Yb/Tm/Gd (18/0.3/x%, x= 0, 10, 20, 40, 60, 81.7)	Extended Data Fig. 5a	Doping concentration of Gd^{3+}
$NaGdF_4$:Yb/Tm (x/0.3%, x = 5, 10, 15, 18, 30)	Extended Data Fig. 5b	Doping concentration of Yb^{3+}
$NaGdF_4$:Yb/Tm (18/x%, x = 0.3, 0.5, 2, 5, 8)	Extended Data Fig. 5c	Doping concentration of Tm^{3+}
$NaGdF_4$:Yb/Tb (12/8%)	Fig. 3h, i	Emission depletion of various Tb-doped nanoparticles
$NaGdF_4$:Yb/Tb (12/8%) @ $NaYF_4$	Fig. 3i	
$NaYF_4$:Yb/Tb (12/8%)	Fig. 3i	

Supplementary Table 2 The parameters used in numerical simulation of optical depletion of upconversion luminescence from $^1G_4$ state in $NaGdF_4$:Yb/Tm (18/0.3%) system assisted with SMED mechanism.

c_1 ($cm^3 s^{-1}$)	c_2 ($cm^3 s^{-1}$)	c_3 ($cm^3 s^{-1}$)	c_4 ($cm^3 s^{-1}$)	w_1 ($cm^3 s^{-1}$)	w_2 ($cm^3 s^{-1}$)
1.3×10^{-18}[a]	1.3×10^{-18}[a]	5.0×10^{-19}[a]	1.5×10^{-18}[a]	1.0×10^{-17}[b]	5.0×10^{-17}[b]
w_3 ($cm^3 s^{-1}$)	w_4 ($cm^3 s^{-1}$)	τ_{Yb1} (s)	τ_1 (s)	τ_3 (s)	τ_5 (s)
4.2×10^{-17}[b]	5.0×10^{-17}[b]	5.0×10^{-4}[c]	3.1×10^{-3}[c]	7.8×10^{-4}[c]	2.5×10^{-4}[c]
τ_6 (s)	τ_7 (s)	β_2 (s^{-1})	β_3 (s^{-1})	β_4 (s^{-1})	β_5 (s^{-1})
6.7×10^{-5}[c]	7.2×10^{-5}[c]	1.7×10^{4}[d]	5.0×10^{3}[d]	1.0×10^{5}[d]	2.0×10^{3}[d]
β_6 (s^{-1})	β_7 (s^{-1})	σ_{p}^{a} (cm^2)	σ_{d}^{a} (cm^2)	σ_{se}^{a} (cm^2)	b_{50}
1.7×10^{3}[d]	1.7×10^{3}[d]	1.5×10^{-20}[e]	6.0×10^{-21}[e]	6.0×10^{-21}[e]	0.65[f]
b_{51}	b_{52}	b_{60}	b_{61}	b_{62}	b_{70}
0.15[f]	0.2[f]	0.57[f]	0.42[f]	0.01[f]	0.09[f]
b_{71}	b_{73}	b_{74}	b_{75}	M_{SEMD} (s^{-1})	
0.58[f]	0.16[f]	0.03[f]	0.14[f]	$0\text{-}2.0\times10^{6}$	

The values of parameters used in the numerical simulation were mainly from our previous work[12] with modification.

[a] Estimated from Ivanova *et al.*[2] and Tkachuk *et al.*[13]

[b] Estimated from Braud *et al.*[14]

[c] Estimated from Ivanova *et al.*[2] and Villanueva-Delgado *et al.*[15]

[d] From Ivanova *et al.*[2]

[e] Estimated from Zhan *et al.*[12]

[f] From Villanueva-Delgado *et al.*[15] with simplification.

Supplementary Table 3 Summarized technical comparison between this work and other reports.

Method	References	FWHM Resolution	FRC Resolution	Saturation intensity, I_{sat}	STED intensity	Pixel Dwell time	SNR	Photobleaching
**SMED**	This work	Average 20 nm	24-32 nm	18.3 kW cm^{-2}	1.09 MW cm^{-2}	0.2 ms	~5	None
**RESOLFT**	*Nature* **478**, 204 (2011)	36 nm	None	Unknow	1 kW cm^{-2}	22 ms	~7.5[a]	50% off, 1200 cycles
**GSD**	*Phys. Rev. Lett.* **98**, 218103 (2007)	80 nm	None	10 kW cm^{-2}	80 kW cm^{-2}	60 ms	Unknow	20% off, 25 cycles
**ESA**	*Angew. Chem. Int. Ed.* **47**, 2685, (2008)	45 nm	None	100 kW cm^{-2}	2.5 MW cm^{-2}	Unknow	Unknow	Severe bleaching for 40 s
**CR-assisted STED**	*Nature* **543**, 229 (2017)	31.2 nm (28nm, deconv.)	None	190 kW cm^{-2}	7.5 MW cm^{-2}	6 ms	~3-4.5[b]	None

[a]The value of SNR was calculated by dividing the peak intensity by the average background noice, which read from the intensity profiles of RESOLFT image of filaments. (Fig. 3c in *Nature* **478**, 204 (2011))
[b]The value of SNR was calculated by dividing the peak intensity by the average background noice, which read from the intensity profiles of STED image of single upconversion nanoparticle. (Fig. 5g,h in *Nature* **543**, 229 (2017))

Supplementary References:

1 Gruber, J. B., Leavitt, R. P. & Morrison, C. A. Absorption spectrum, energy levels, and crystal-field parameters of Tm^{3+}:$LaCl_3$. *J. Chem. Phys.* **74**, 2705-2709 (1981).

2 Ivanova, S. E., Tkachuk, A. M., Mirzaeva, A. & Pellé, F. Spectroscopic study of thulium-activated double sodium yttrium fluoride $Na_{0.4}Y_{0.6}F_{2.2}$:Tm^{3+} crystals: I. Intensity of spectra and luminescence kinetics. *Opt. Spectrosc.* **105**, 228-241 (2008).

3 Gao, P., Prunsche, B., Zhou, L., Nienhaus, K. & Nienhaus, G. U. Background suppression in fluorescence nanoscopy with stimulated emission double depletion. *Nat. Photon.* **11**, 163-169 (2017).

4 Ma, Y. & Ha, T. Fight against background noise in stimulated emission depletion nanoscopy. *Phys. Biol.* **16**, 051002 (2019).

5 Vicidomini, G., Moneron, G., Eggeling, C., Rittweger, E. & Hell, S. W. STED with wavelengths closer to the emission maximum. *Opt. Express* **20**, 5225-5236 (2012).

6 Wegh, R. T., Donker, H., Oskam, K. D. & Meijerink, A. Visible Quantum Cutting in $LiGdF_4$:Eu^{3+} Through Downconversion. *Science* **283**, 663 (1999).

7 Wang, F. *et al.* Tuning upconversion through energy migration in core–shell nanoparticles. *Nat. Mater.* **10**, 968-973 (2011).

8 Würth, C., Fischer, S., Grauel, B., Alivisatos, A. P. & Resch-Genger, U. Quantum Yields, Surface Quenching, and Passivation Efficiency for Ultrasmall Core/Shell Upconverting Nanoparticles. *J. Am. Chem. Soc.* **140**, 4922-4928 (2018).

9 Zhang, H., Li, Y., Lin, Y., Huang, Y. & Duan, X. Composition tuning the upconversion emission in $NaYF_4$:Yb/Tm hexaplate nanocrystals. *Nanoscale* **3**, 963-966 (2011).

10 Wang, G. *et al.* Intense ultraviolet upconversion luminescence from hexagonal $NaYF_4$:Yb^{3+}/Tm^{3+} microcrystals. *Opt. Express* **16**, 11907-11914 (2008).

11 Bestvater, F. *et al.* Two-photon fluorescence absorption and emission spectra of dyes relevant for cell imaging. *J. Microsc. - Oxford* **208**, 108-115 (2002).

12 Zhan, Q. *et al.* Achieving high-efficiency emission depletion nanoscopy by employing cross relaxation in upconversion nanoparticles. *Nat. Commun.* **8**, 1058 (2017).

13 Tkachuk, A. M., Razumova, I. K., Perlin, E. Y., Joubert, M. F. & Moncorge, R. Luminescence self-quenching in Tm^{3+}: YLF crystals: II. The luminescence decay and macrorates of energy transfer. *Opt. Spectrosc.* **90**, 78-88 (2001).

14 Braud, A. *et al.* Energy-transfer processes in Yb:Tm-doped KY_3F_{10}, $LiYF_4$, BaY_2F_8 single crystals for laser operation at 1.5 and 2.3 μm. *Phys. Rev. B* **61**, 5280-5292 (2000).

15 Villanueva-Delgado, P., Biner, D. & Krämer, K. W. Judd–Ofelt analysis of β-$NaGdF_4$: Yb^{3+}, Tm^{3+} and β-$NaGdF_4$:Er^{3+} single crystals. *J. Lumin.* **189**, 84-90 (2017).
