# Supplementary material for: Super-resolution microscopy enabled by high-efficiency surface-migration emission depletion
Source: Nat Commun. 2022 Nov 4;13:6636. doi: 10.1038/s41467-022-33726-7 (PMC9636245; doi:10.1038/s41467-022-33726-7)
Supplement: Supplementary file 2 — Reporting Summary [file 41467_2022_33726_MOESM2_ESM.pdf]

nature portfolio

Corresponding author(s): Xiaogang LIU, Qiuqiang ZHAN

Last updated by author(s): Aug 5, 2022

Reporting Summary

Nature Portfolio wishes to improve the reproducibility of the work that we publish. This form provides structure for consistency and transparency in reporting. For further information on Nature Portfolio policies, see our Editorial Policies and the Editorial Policy Checklist.

Statistics

For all statistical analyses, confirm that the following items are present in the figure legend, table legend, main text, or Methods section.

n/a	Confirmed	
☐	☒	The exact sample size (n) for each experimental group/condition, given as a discrete number and unit of measurement
☐	☒	A statement on whether measurements were taken from distinct samples or whether the same sample was measured repeatedly
☒	☐	The statistical test(s) used AND whether they are one- or two-sided<br>*Only common tests should be described solely by name; describe more complex techniques in the Methods section.*
☐	☒	A description of all covariates tested
☐	☒	A description of any assumptions or corrections, such as tests of normality and adjustment for multiple comparisons
☐	☒	A full description of the statistical parameters including central tendency (e.g. means) or other basic estimates (e.g. regression coefficient) AND variation (e.g. standard deviation) or associated estimates of uncertainty (e.g. confidence intervals)
☒	☐	For null hypothesis testing, the test statistic (e.g. F, t, r) with confidence intervals, effect sizes, degrees of freedom and P value noted<br>*Give P values as exact values whenever suitable.*
☒	☐	For Bayesian analysis, information on the choice of priors and Markov chain Monte Carlo settings
☒	☐	For hierarchical and complex designs, identification of the appropriate level for tests and full reporting of outcomes
☒	☐	Estimates of effect sizes (e.g. Cohen's d, Pearson's r), indicating how they were calculated

Our web collection on statistics for biologists contains articles on many of the points above.

Software and code

Policy information about availability of computer code

Data collection	Software Olympus FV10 ASW ver. 4.0a was used to control laser scanning microscopy imaging and acquire images. Ocean Optics Software (Product Version: SpectraSuite) was used to control spectrometer and acquire spectra. No custom algorithms or software that were central to the data collection.
Data analysis	Matlab (v2019a) was used for the analysis of spectra, the analysis of microscopic images and the calculation of numerical simulations. FRC analysis was performed with the code provided by Nat. Methods 16, 175–178 (2019).The first principles calculations based on DFT were performed using the Vienna ab initio simulation package with the projector augmented wave method. The codes for theoretical modelling and numerical simulations are available from the corresponding authors on request.

For manuscripts utilizing custom algorithms or software that are central to the research but not yet described in published literature, software must be made available to editors and reviewers. We strongly encourage code deposition in a community repository (e.g. GitHub). See the Nature Portfolio guidelines for submitting code & software for further information.

Data

Policy information about availability of data

All manuscripts must include a data availability statement. This statement should provide the following information, where applicable:

- Accession codes, unique identifiers, or web links for publicly available datasets
- A description of any restrictions on data availability
- For clinical datasets or third party data, please ensure that the statement adheres to our policy

The data generated in this study are provided in the Source data files. Additional data are available from the corresponding authors upon request.

Human research participants

Policy information about studies involving human research participants and Sex and Gender in Research.

Reporting on sex and gender	N/A
Population characteristics	N/A
Recruitment	N/A
Ethics oversight	N/A

Note that full information on the approval of the study protocol must also be provided in the manuscript.

Field-specific reporting

Please select the one below that is the best fit for your research. If you are not sure, read the appropriate sections before making your selection.

☒ Life sciences ☐ Behavioural & social sciences ☐ Ecological, evolutionary & environmental sciences

For a reference copy of the document with all sections, see nature.com/documents/nr-reporting-summary-flat.pdf

Life sciences study design

All studies must disclose on these points even when the disclosure is negative.

Sample size	No statistical method was used to predetermine the sample size. For the experiments of cell imaging, we acquired images of several cells from different areas of the 96-well plates and similar results were obtained among different areas. The whole experiment has been repeated at least five times during our study. For the single-particle imaging, multiple sample slides were prepared, and single particles in different areas of the slides showed similar imaging results and resolution enhancement effect. For the spectroscopy study, the results were collected from different sample points and were averaged, which can reflected the overall luminescence properties of the studied samples. Data were collected from three independent measurement.
Data exclusions	No data are excluded from the analyses.
Replication	All attempts at replication were successful. The experiments were replicated at least five times independently and similar results were obtained.
Randomization	Randomization is not relevant to our study as there was no specific allocation to experimental groups in the study.
Blinding	Blinding was not performed, since it is not applicable to this type of study. The cells were labeled by the samples and directly used for imaging experiments.

Reporting for specific materials, systems and methods

We require information from authors about some types of materials, experimental systems and methods used in many studies. Here, indicate whether each material, system or method listed is relevant to your study. If you are not sure if a list item applies to your research, read the appropriate section before selecting a response.

Materials & experimental systems

n/a	Involved in the study
☒	☐ Antibodies
☐	☒ Eukaryotic cell lines
☒	☐ Palaeontology and archaeology
☒	☐ Animals and other organisms
☒	☐ Clinical data
☒	☐ Dual use research of concern

Methods

n/a	Involved in the study
☒	☐ ChIP-seq
☒	☐ Flow cytometry
☒	☐ MRI-based neuroimaging

Eukaryotic cell lines

Policy information about cell lines and Sex and Gender in Research

Cell line source(s)	HeLa cell line was provided by Laboratory Animal Center, Sun Yat-Sen University (Guangzhou, China)
Authentication	None of the cell lines used were authenticated.
Mycoplasma contamination	The cell lines were not tested for mycoplasma contamination.
Commonly misidentified lines (See ICLAC register)	None.
